# Comparative Genomics of Members of the Genus *Defluviicoccus* With Insights Into Their Ecophysiological Importance

**DOI:** 10.3389/fmicb.2022.834906

**Published:** 2022-04-12

**Authors:** Irina Bessarab, Abdul Majid Maszenan, Mindia A. S. Haryono, Krithika Arumugam, Nay Min Min Thaw Saw, Robert J. Seviour, Rohan B. H. Williams

**Affiliations:** ^1^Singapore Centre for Environmental Life Sciences Engineering, National University of Singapore, Singapore, Singapore; ^2^Nanyang Environment & Water Research Institute (NEWRI), Nanyang Technological University, Singapore, Singapore; ^3^NUS Environmental Research Institute, National University of Singapore, Singapore, Singapore; ^4^Singapore Centre for Environmental Life Sciences Engineering, Nanyang Technological University, Singapore, Singapore; ^5^School of Life Sciences, La Trobe University, Melbourne, VIC, Australia

**Keywords:** activated sludge, glycogen accumulating organisms, *Defluviicoccus*, *Defluviicoccus vanus*, enhanced biological phosphorus removal (EBPR)

## Abstract

Members of the genus *Defluviicoccus* occur often at high abundances in activated sludge wastewater treatment plants designed to remove phosphorus, where biomass is subjected to alternating anaerobic feed/aerobic famine conditions, believed to favor the proliferation of organisms like *Ca*. Accumulibacter and other phosphate-accumulating organisms (PAO), and *Defluviicoccus.* All have a capacity to assimilate readily metabolizable substrates and store them intracellularly during the anaerobic feed stage so that under the subsequent famine aerobic stage, these can be used to synthesize polyphosphate reserves by the PAO and glycogen by *Defluviicoccus.* Consequently, *Defluviicoccus* is described as a glycogen-accumulating organism or GAO. Because they share a similar anaerobic phenotype, it has been proposed that at high *Defluviicoccus* abundance, the PAO are out-competed for assimilable metabolites anaerobically, and hence aerobic P removal capacity is reduced. Several *Defluviicoccus* whole genome sequences have been published (*Ca.* Defluviicoccus tetraformis, *Defluviicoccus* GAO-HK, and *Ca*. Defluviicoccus seviourii). The available genomic data of these suggest marked metabolic differences between them, some of which have ecophysiological implications. Here, we describe the whole genome sequence of the type strain *Defluviicoccus vanus^T^*, the only cultured member of this genus, and a detailed comparative re-examination of all extant *Defluviicoccus* genomes. Each, with one exception, which appears not to be a member of this genus, contains the genes expected of GAO members, in possessing multiple copies of those for glycogen biosynthesis and catabolism, and anaerobic polyhydroxyalkanoate (PHA) synthesis. Both 16S rRNA and genome sequence data suggest that the current recognition of four clades is insufficient to embrace their phylogenetic biodiversity, but do not support the view that they should be re-classified into families other than their existing location in the *Rhodospirillaceae.* As expected, considerable variations were seen in the presence and numbers of genes encoding properties associated with key substrate assimilation and metabolic pathways. Two genomes also carried the *pit* gene for synthesis of the low-affinity phosphate transport protein, pit, considered by many to distinguish all PAO from GAO. The data re-emphasize the risks associated with extrapolating the data generated from a single *Defluviicoccus* population to embrace all members of that genus.

## Introduction

All continuous flow enhanced biological phosphorus removal wastewater treatment plants (EBPR) operate by passing the biomass continuously between anaerobic and aerobic reactors, a plant configuration considered essential for removing phosphorus (P) microbiologically by advantaging selectively the populations involved in its removal (Seviour and McIlroy, 2008; Stokholm-Bjerregaard et al., 2017; Nielsen et al., 2019; Roy et al., 2021). One popular but not universally accepted explanation as to why EBPR plants often perform badly is that the polyphosphate-accumulating organisms (PAO) are out-competed in the EBPR anaerobic “feed” stage by other bacteria known collectively as the glycogen-accumulating organisms (GAO) (Cech and Hartman, 1993; Liu et al., 1996; Law et al., 2016; Nielsen et al., 2019). These populations share with the PAO a similar anaerobic phenotype in being able to assimilate readily metabolizable substrates present in the plant influent, which are used to synthesize intracellular storage compounds of a diverse chemical composition. Intracellular polyphosphate (polyP) reserves, an anaerobic TCA cycle, and glycogen are used by the PAO as anaerobic sources of energy and reducing power for both substrate assimilation and subsequent intracellular biosynthesis of storage products (McMahon et al., 2010; Zhou et al., 2010), and orthophosphate is released into the bulk liquid. Storage compounds varying in their chemical composition (see below) are then reutilized in the subsequent aerobic “famine” stage, thus providing energy to support PAO growth, the assimilation of phosphate from the bulk liquid and subsequent synthesis of polyP granules as energy stores needed to support their subsequent anaerobic metabolism (Oehmen et al., 2007; Seviour and McIlroy, 2008). Stored P is then removed from the system by sludge wasting.

The GAO also assimilate metabolites under anaerobic conditions, but now, in the absence of polyphosphate reserves, use the stored glycogen as their main source of energy and reducing power from the activity of the Embden Meyerhof Parnas (EMP) pathway. As with the PAO, this storage material is then metabolized to support GAO growth under subsequent aerobic conditions, and to synthesize and store glycogen for use during the subsequent anaerobic phase. Hence, as no intracellular polyP is synthesized and stored intracellularly, then at high abundances, the GAO are thought to reduce EBPR capacity (Oehmen et al., 2007, 2010; Mielczarek et al., 2013; Nielsen et al., 2019). However, emerging data from full-scale systems suggest that any PAO–GAO competition occurs primarily in ecological niches generated by PAO enrichment protocols (Law et al., 2016; Nielsen et al., 2019), highlighting the need to maintain a broader view of the ecophysiological impact of GAO populations on EBPR capacity.

The GAO phenotype is found in phylogenetically diverse bacterial populations. These include the gammaproteobacterial *Ca*. Competibacter (Kong et al., 2006) and *Ca.* Contendobacter (McIlroy et al., 2014, 2015), the betaproteobacterial *Ca*. Propionivibrio aalborgensis (Albertsen et al., 2016) and Spb280 (Kong et al., 2007), the actinobacterial *Micropruina glycogenica* (Shintani et al., 2000; McIlroy et al., 2018), and the alphaproteobacterial *Defluviicoccus vanus* (Maszenan et al., 2005). Of these, only *M. glycogenica* and *D. vanus* have been cultured. As with the known PAO, considerable metabolic diversity exists between individual GAO populations. Thus, while *M. glycogenica* utilize and store sugars and amino acids generated by fermentation, *Ca*. Propionivibrio aalborgensis (Albertsen et al., 2016), an unusually aerobic member of this genus and closely related phylogenetically to the PAO *Ca*. Accumulibacter phosphatis (Stokholm-Bjerregaard et al., 2017), possesses the same anaerobic phenotype as this PAO, in assimilating short-chain fatty acids and storing them as poly-ß-hydroxy alkanoates (PHA). This same anaerobic phenotype is seen in *Ca* Competibacter and *Ca.* Contendobacter (McIlroy et al., 2014) and all members of the genus *Defluviicoccus* examined to date (Nittami et al., 2009; Nobu et al., 2014; Wang et al., 2014; Stokholm-Bjerregaard et al., 2017; Onetto et al., 2019).

**FIGURE 1:**
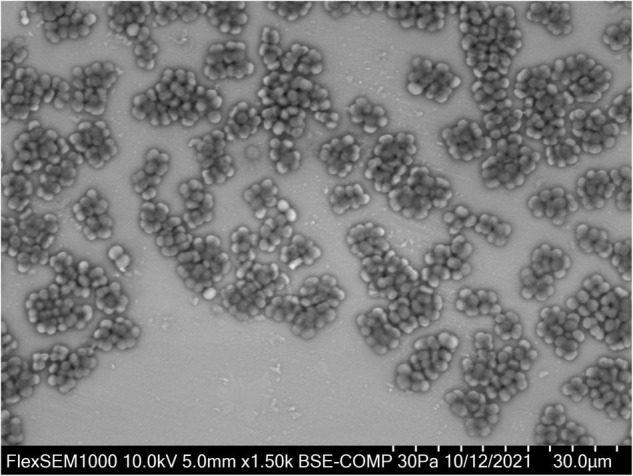
*Defluviicoccus vanus* showing its distinctive cocci arranged in tetrads, and clusters of tetrads. For VP-SEM imaging, the *D. vanus* cells were grown on GS agar for 3 weeks. Individual colonies were selected and transferred onto a glass slide cut to the size of the specimen stub. Samples were visualized uncoated in a variable-pressure scanning electron microscope VP-SEM (Hitachi FlexSEM 1000 II) at 30 Pa and accelerating voltage of 10 kV, using a BSE detector.

Based on 16S rRNA sequence data, *Defluviicoccus* is placed currently in the family *Rhodospirillaceae* in the order *Rhodospirillales* (Maszenan et al., 2005), where members of this genus fall at present into four clades (Wong et al., 2004; Meyer et al., 2006; Burow et al., 2007; McIlroy and Seviour, 2009; Nittami et al., 2009). However, this phylogenetic diversity has not always been recognized in many *Defluviicoccus* publications based on partial 16S rRNA amplicon sequencing, and in the absence of any accompanying FISH analyses. *D. vanus^T^*, the type species, which was isolated from an EBPR wastewater treatment plant (WWTP) treating mainly brewery wastes in Pilsen, Czechia showing poor P removal capacity (Maszenan et al., 2005), is a member of clade 1, as is *Ca. Defluviicoccus* tetraformis (Nobu et al., 2014), while *Defluviicoccus* strain GAO-HK (Wang et al., 2014) belongs to clade II. These and clade IV (McIlroy and Seviour, 2009) members of this genus, and visualized *in situ* with clade targeted FISH probes, have the characteristic morphology of cocci arranged in tetrads (Figure 1) (McIlroy and Seviour, 2009; McIlroy et al., 2011). However, *Ca.* Defluviicoccus seviourii, a member of clade III, is filamentous (Nittami et al., 2009), sharing the distinctive *Nostocoida limicola II* morphotype described by Liu et al. (2000) and Seviour et al. (2002).

Draft genomes of *Defluviicoccus* strain GAO-HK, *Ca.* D. tetraformis, *Ca*. D. seviourii, and SSA4 have been generated from metagenome assemblies (Nobu et al., 2014; Wang et al., 2014; Arumugam et al., 2019, 2021; Onetto et al., 2019), and in general support the earlier metabolic models of Lopez-Vazquez et al. (2009) and Oehmen et al. (2010). They also confirm the presence of many of the metabolic processes detailed in earlier physiological and metabolic inhibitor studies of members of this genus (Saunders et al., 2007; Burow et al., 2008a,b, 2009). Furthermore, each genome harbors the genes encoding the same pathways expected of GAO associated with carbon recycling, involving glycogen and PHA synthesis and their degradation. These early genomic sequencing data also revealed several ecologically important variations among them, the most notable being a lack of the glyoxylate cycle pathway in *Ca.* D. *seviourii*, which instead possessed the anaplerotic ethylmalonyl CoA pathway (Onetto et al., 2019). It alone of the three was thought to have the gene encoding a Na/glutamate symporter, while a gene (*pit*) encoding a low-affinity phosphate transporter pit was seen only in the genome of strain GAO-HK (clade II) (Nobu et al., 2014; Wang et al., 2014; Onetto et al., 2019).

Consequently, we undertook to sequence the genome of the type strain *D. vanus^T^*, and to compare it to all available genomes of other uncultured *Defluviicoccus* populations. The aim was to understand better their ecophysiology and expose the genomic differences existing among them, especially those of potential ecological importance.

## Materials and Methods

### Culture Revival, DNA Extraction, and Identification Confirmation

*Defluviicoccus vanus* (strain Ben 114*^T^*) was obtained from the NCIMB (National Collection of Industrial, Food and Marine Bacteria, Aberdeen, United Kingdom) culture deposited there in 2003. The freeze-dried culture was revived in modified glucose sulfite (GS) agar (Williams and Unz, 1985; Maszenan, 2001) according to the culture revival procedure recommended by NCIMB. After ca. 3 weeks of incubation at 25°C, cells with the distinctive tetrad morphology expected of strain Ben 114*^T^* were seen (Figure 1). The strain was stored on GS agar at 4°C and maintained at 25°C. DNA was extracted from *D. vanus* cells grown on solid media. For DNA extraction, the biomass was collected from the GS agar plates with sterile GS medium (Williams and Unz, 1985), pelleted by centrifugation and washed 1–2× with sterile PBS. Washed biomass was pre-treated either with Proteinase K (Sigma, >10 mg/ml) at 55°C for 30 min followed by 80°C for 10 min, or with MetaPolyzyme (Sigma) at 37°C for 4–6 h. Total DNA was extracted from pre-treated biomass using two methods: FastDNA™ SPIN kit for soil (MP Biomedicals), using Lysing Matrix E and 2 × bead beating with a FastPrep homogenizer (MP Biomedicals), or with DNeasy PowerBiofilm kit (Qiagen, Hilden, Germany) according to the manufacturer’s protocols. To confirm that the revived culture was *D. vanus* strain Ben 114*^T^*, a nearly full-length 16S rRNA gene sequence was generated by PCR amplification of extracted genomic DNA with primers 27F (5′-AGAGTTTCMTGGCTCAG-3′) and 1429R (5′-TACGGYTACCTTGTTACGACTT-3′) and KapaHiFi Hot Start Ready Mix (Roche Sequencing, Pleasanton, CA, United States). The nucleotide sequence of the PCR product was determined by Sanger sequencing (ABI PRISM 3500 Genetic Analyzer, Applied Biosystems, Waltham, MA, United States). The paired-end ABI-Sanger PCR products were trimmed and merged using SeqMan NGen (version 17.0.22 with default parameters), and Sanger Sequence Assembly Software from DNASTAR (14-day trial version). The merged reads were annotated against SILVA database (SSURef_NR99_132_SILVA_13_12_17_opt.arb) (Quast et al., 2013) using sina-1.6.0-linux running default settings except -t -v –log-file –meta-fmt csv, and with –lca-fields set for all five databases, namely, tax_slv, tax_embl, tax_ltp, tax_gg, and tax_rdp.

### Long Read Genome Sequencing and Data Analyses

To obtain the complete genome sequence of *D. vanus^T^*, nanopore sequencing was performed on a MinION Mk1B instrument (Oxford Nanopore Technologies) using a SpotON FLO MIN106 FAK45997 flow cell with R9.4 chemistry. A sequencing library was constructed from 400 ng of *D. vanus* genomic DNA using Rapid Barcoding Sequencing Kits SQK-RBK004 supplied by Oxford Nanopore Technologies, with two barcodes to differentiate gDNA obtained with the different extraction protocols. The sequencing run was operated for approximately 48 h. Data acquisition was performed using MinKNOW software (release 19.06.7) without live base calling on an HP ProDesk 600G2 computer (64-bit, 16 GB RAM, 2 Tb SSD HD). Long reads were base called using guppy (CPU version 3.2.1) and the base called reads were trimmed for adaptors using Porechop (version 0.2.2) with default settings except -v 3 -t 20. Long reads were assembled using Unicycler (version 0.4.7) (Wick et al., 2017) with default settings except -t 40 –keep 3. We refer to assembled sequences here as *long read assembled contigs* (LRAC). DIAMOND (version 0.9.24) (Buchfink et al., 2015, 2021) was used to perform alignment of LRAC sequences (with default settings except -f 100 -p 40 -v –log –long-reads -c1 -b12) against the NBCI-NR database (February, 2019). From the MEGAN Community Edition suite (version 6.17.0), Daa-meganizer was used to format the .daa output file for use in the MEGAN GUI (version 6.17.0) (Huson et al., 2018). Within MEGAN, LRAC sequences were exported with the “Export Frame-Shift Corrected Reads” option to obtain frameshift corrected sequences. The frameshift corrected LRAC sequences were annotated using Prokka version 1.13 (Seemann, 2014) with default settings except –debug –addgenes –rfam. Genome quality statistics were obtained using CheckM (version 1.0.11; Parks et al., 2015).

Coverage profiles were generated from long read data against the LRAC sequences using minimap2 (version 2.17) with the following flags -ax map-ont. Sorted .bam files were processed subsequently using bedtools genomeCoverageBed (version 2.26.0) (Quinlan and Hall, 2010) with the following flags -d. Alignments to the genome sequences were then examined using the Integrated Genome Viewer (IGV version 2.4.14) (Robinson et al., 2011) to evaluate genome integrity and to identify the presence of any misassembled regions.

16S-SSU rRNA genes were identified using the Prokka workflow and annotated against SILVA database (SURef_NR99_132_SILVA_13_12_17_opt.arb) (Quast et al., 2013) using sina-1.6.0-linux running default settings except -t -v –log-file –meta-fmt csv and with –lca-fields set for all five databases, namely, tax_slv, tax_embl, tax_ltp, tax_gg, and tax_rdp.

### Short Read Sequencing and Data Analyses

Genomic DNA Library preparation was performed using a modified version of the Illumina TruSeq DNA Sample Preparation protocol. A MiSeq sequencing run was then performed with a read length of 301 bp (paired-end). The raw FASTQ files were processed with cutadapt (version 2.5) and the following arguments: –overlap 10 -m 30 -q 20,20 –quality-base 33. Reads were assembled using SPAdes (version 3.13.0, executed with default parameters except -k 21,33,55,77,99,127 –meta -t 44) (Bankevich et al., 2012). The SPAdes contig fasta file was processed using the R package RKXM^1^ and the chromosomal genome was manually binned in the GC-coverage plane. Genome quality statistics were obtained again using CheckM (version 1.0.11) (Parks et al., 2015). The concordance statistic was computed between contigs in short read assembly and the long read assembled chromosome using the R package srac2lrac (Arumugam et al., 2019, 2021). Coverage profiles of short read data against the long read assembly was achieved using the same methods described in the immediately preceding section, with minimap2 settings -ax sr -a -t 20.

### Genomes of Other Members of the Genus *Defluviicoccus* Used in the Study

The following *Defluviicoccus* genomes were examined in this study: (1) the draft genome of clade III *Ca.* Defluviicoccus seviourii from Onetto et al. (2019); (2) the draft genome of *Defluviicoccus* clade II GAO-HK obtained by Wang et al. (2014); (3) the draft genome of clade I *Ca.* D. tetraformis strain TFO71 of Nobu et al. (2014); (*4*) the draft genome attributed to *Defluviicoccus* obtained by Slaby et al. (2017), and of uncertain classification (see later), and denoted as bin 129 in that paper; (5) a *Defluviicoccus* genome obtained previously by us from long read metagenome data from a PAO enrichment reactor (Arumugam et al., 2019, 2021), denoted as *Defluviicoccus* sp. SSA4; and (6) three long read metagenome-assembled genomes from members of the genus *Defluviicoccus* recovered by Singleton et al. (2021), and denoted here as *Defluviicoccus* FRED MAXAC 307, *Defluviicoccus* FRED MAXAC 378 and *Defluviicoccus* KALU MAXAC 148. The PRM01 genome of Onetto et al. (2019) was excluded from this study because of its high levels of sequence contamination (Onetto et al., 2019). We computed average nucleotide identity (ANI) value comparisons between these genomes using FastANI (Jain et al., 2018).

### Genome Annotation

All genome assemblies were annotated using Prokka version 1.13 default settings except –debug –addgenes –rfam (Seemann, 2014). To document the presence or absence of canonical metabolic pathways, translated gene sequences from the Prokka (.faa files) were put into the KEGG Mapper Reconstruction workflow of the BlastKOALA webserver (Kanehisa et al., 2016) selecting KEGG Modules with the “including any incomplete” option, in order to fully document the degree of completeness of each selected pathway. The following nomenclature was used to describe the presence and completeness of pathways; (1) pathways labeled with “C” are considered complete, and classified as such by BlastKOALA, or determined to be complete from additional manual annotation (see below); (2) pathways largely intact (no more than 2 missing blocks as defined by BlastKOALA) are labeled with “+”; (3) pathways classified by BlastKOALA as incomplete or missing more than 2 blocks are labeled with “?”; and (4) pathways for which no gene products could be identified in the genome are labeled with “–”.

In the case of key pathways that were classified as largely intact (category 2 above), the possibility that partial incompleteness had resulted from false-negative annotations of individual gene products was examined by manually cross-referencing them against annotations available from Prokka. Any ambiguities of gene-product annotation were tested using the NCBI blastp webserver searching against the BLAST nr database. All annotation data are provided as Supplementary Data Files as tab-delimited text files combining gene-product level annotations from Prokka and BlastKOALA (Supplementary Data File 1).

### Comparative Analysis of 16S-SSU rRNA Genes

All 16S-SSU rRNA sequences annotated to members of genus *Defluviicoccus* (*n* = 83) from the SILVA database (version 132) (Quast et al., 2013) were downloaded and combined with the 1116S-SSU rRNA sequences harbored on contigs from each of the draft genomes listed earlier.

For testing hypotheses about the taxonomic placement of these *Defluviicoccus* populations, full-length 16S SSU-rRNA sequences were downloaded from the ‘The All-Species Living Tree’ Project (LTP) (file: LTP_04_2021_compressed.fasta from the download page^2^). From this database, all sequences annotated to members of *Rhodospirillales* (*n* = 344), which include the existing sequence from *D. vanus*^T^** and two sequences from members of the genus *Tistrella* (*Tistrella bauzanesis* and *Tistrella mobilis*) were augmented with the set of 11 16S sequences from the nine *Defluviicoccus* genomes listed above and a set of 19 16S sequences annotated to the order *Tistrellales* derived from cultured isolates.

All subsequent phylogenetic analyses were conducted with the SILVA ACT webserver^3^ (Pruesse et al., 2012) using RAxML with “Model to use” set to GTR and “Rate model for likelihoods” set to “Gamma” (settings as available within the “Compute tree” option, with “*Denovo* with user sequences only” selected). The output .tree file was visualized using the plot .phylo function in the R library *ape* (Paradis and Schliep, 2019). The output.*in_fasta* files were imported into R using the read .alignment function in the R package seqinr, and converted to character matrices using the R/seqinr function as .matrix.alignment. The percent (sequence) identity (PID) was computed for each pairwise combination of sequences using a custom R script that implemented PID as the number of position-wide identical nucleotides divided by the total number of aligned nucleotides, not counting the occurrence of shared indels.

### Comparative Analyses of *Defluviicoccus* Genomes

Phylogenetic analyses based on whole genome sequences were conducted with GTDB-Tk v0.3.2 (Parks et al., 2018, 2020; Chaumeil et al., 2019) using the *de novo* workflow and 120 concatenated-marker genes selective for bacteria. In addition to the nine *Defluviicoccus* draft genomes (Table 1), these analyses included other members of the *Rhodospirillales* (using the following arguments –taxa_filter o__*Rhodospirillales*, o__*Rhodospirillales*_A, o__*Rhodospirillales*_B), and following Onetto et al. (2019), the genomes from members of the genus *Gemmatimonas* were assigned as the outgroup taxa (using the argument –outgroup_taxon g__*Gemmatimonas*).

**TABLE 1:** Properties of *Defluviicoccus* genomes examined in this study.

Property	*D. vanus*	*Ca. D. tetraformis TF071*	*Ca. D.* sp. *SSA4*	*Ca. D.* sp. *Fred MAXAC 307*	*Ca. D.* sp. *Fred MAXAC 378*	*Ca. D.* sp. *GAO-HK*	*Ca. D. seviourii*	*Ca. D* sp. *Kalu MAXAC 148*	*Ca. D.* sp. *bin 129*
Total sequence length (bp)	4.16e6	4.64e6	4.35e6	4.28e6	4.31e6	3.98e6	3.29e6	3.80e6	4.83e6
Number of contigs	1	162	1	11	45	605	54	27	122
N50 (bp)	4.16e6	7.21e4	4.35e6	2.67e6	1.80e5	1.20e4	1.40e5	4.32e5	5.68e4
GC (%)	63.56	65.29	63.60	63.70	64.04	66.06	64.83	65.93	69.54
Completeness (%)	95.48	97.76	94.64	97.04	91.94	94.19	98.01	93.65	86.57
Contamination (%)	0.00	0.17	0.50	0.75	1.74	1.24	0.00	0.00	0.00
Strain heterogeneity	0.00	0.00	0.00	50.00	0.00	33.33	0.00	0.00	0.00
Number of ORF	3764	4090	4225	3883	4594	3844	2990	3672	4628
Number of rRNA genes	6	3	3	6	5	2	6	3	1
Number of tRNA genes	51	45	46	54	45	44	50	47	45

### Data Availability

Raw sequence data from both long and short read sequencing are available from NCBI Short Read Archive *via* BioProject accession PRJNA635277. The *D*. *vanus^T^* chromosomal and plasmid genomes are available *via* GenBank accessions CP053923.1 and CP053924.1, respectively.

## Results and Discussion

### Genome Assembly, Annotation, and Overview of Gene Level Annotation for *D. vanus^T^*

**FIGURE 2:**
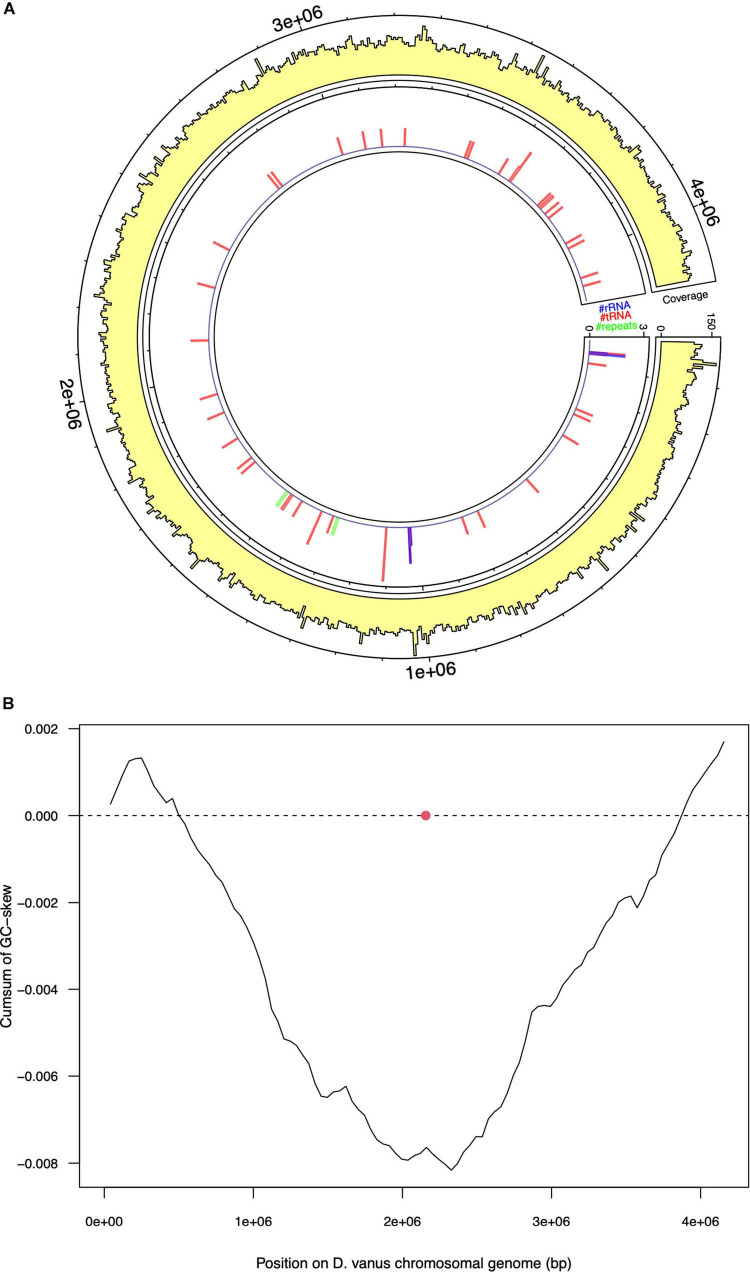
Selected features of the structure and composition of the *D. vanus* chromosomal genome. **(A)** Circular visualization of the *D. vanus* chromosomal genome. The outmost tracks show the mean coverage estimated from mapping long reads to the genome, with per-base coverage binned into 5-kbp non-overlapping windows; the innermost track shows positions of rRNA genes (blue), tRNA genes (blue), and repeat regions (green) with counts obtained within the same 5-kbp windows as above. **(B)** Cumulative GC-plots showing the origin and terminus of replication, with the red closed circle showing the genomic position of the chromosomal replication initiator protein (*dnaA*) gene (CDGBEKEE_02056; Supplementary Data File 1).

Assembly of the long read genome sequence data from *D. vanus^T^* yielded two circular contigs. The longer contig was 4.1 Mbp in length (coverage 100× from long read data, and 800× from short read). The other was 70 kbp in length (coverage 30× from long read and 150× from short read data) and is discussed below. The longer contig met the MIMAG criteria (Bowers et al., 2017) of a high-quality genome, as supported by CheckM-derived completeness and contamination values, observed as 95.48 and 0.0%, respectively. A total of 3,764 protein-encoding genes, including two complete ribosomal RNA gene operons and 51 tRNA encoding genes, 1 tmRNA gene, and 86 miscellaneous RNA genes were identified, together with four repeat regions associated with CRISPR repeat sequences (Table 1 and Figure 2A). The structure of the cumulative GC plot (Grigoriev, 1998) was consistent with the presence of a single replication origin (Figure 2B).

Annotation of the entire contig sequence with GTDB-Tk and individual 16S rRNA encoding genes (see further analyses below), using SILVA, confirmed its placement to the genus *Defluviicoccus*. Based on these data, we conclude that the longer contig is the chromosomal genome sequence of *D. vanus^T^*, which showed a high degree of similarity to the draft genome recovered from the short read sequence obtained from the same DNA aliquot (Supplementary Figure 1), with the concordance (κ) statistic holding a value of 0.98 (Arumugam et al., 2019, 2021) (Supplementary Figure 2).

Of the 3,764 protein encoding genes on the chromosomal genome, 1,989 (52.8%) were assigned a functional annotation by Prokka, with the remaining 1,775 (47.2%) classified as hypothetical proteins. From a separate analysis against KEGG, 2,476/3,764 genes (65.8%) were assigned a KEGG Orthology (KO) annotation (representing 1,642 unique KO identifiers) and 386 were annotated to 299 unique KO identifiers that were members of 139 KEGG Modules (Supplementary Data File 1).

The shorter contig was hypothesized to be a non-chromosomal replicon, because of its short length and circularity. A total of 67 protein-coding genes were detected on this shorter contig, of which 10 were classified as hypothetical proteins. All 67 were annotated to a KEGG Orthology identifier but with no KEGG Modules being implicated (Supplementary Data File 2). The contig contained gene modules known to be associated with replication (*rep*, *par*) and propagation (Type IV secretion system), and consequently is most probably a conjugative plasmid (Norman et al., 2009). Adaptation modules encoding various metal resistance systems, including a complete *czc* operon were observed. BLAST nucleotide analysis of this plasmid genome showed limited sequence similarity to any sequences in NCBI nt (5.8% query cover on the top ranked subject sequence; see Supplementary Data File 3), suggesting that it had not been described previously.

### Phylogenetic Analyses of *D. vanus*^T^** and Other *Defluviicoccus* Genomes Using 16S rRNA and Whole Genome Sequences

**FIGURE 3:**
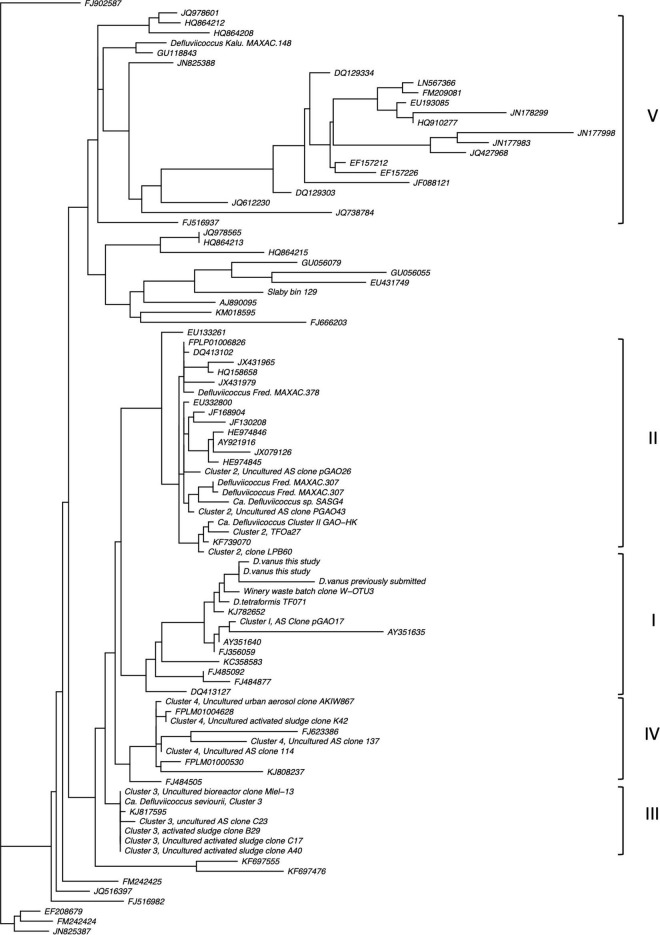
Ribosomal SSU-rRNA gene phylogram showing clusters containing members of the genus *Defluviicoccus*. Note the recapitulation of previously defined Cluster groups (I–IV) as well as evidence of a new cluster (V) associated typified by 16S rRNA sequences from the sample from Kalundborg WWTP (for further details, see Section “Materials and Methods: Comparative Analysis of 16S-SSU rRNA Genes”).

The topology of the 16S rRNA tree constructed from sequences from these nine *Defluviicoccus* showed the expected pattern groupings of previously identified clades I–IV members (Figure 3), with the 16S rRNA gene from the recovered *D. vanus*^T^** genome being closest to those recovered earlier from this isolate (Maszenan et al., 2005). *D. vanus*^T^** fell into the same clade, Clade 1, as did *Ca*. D. tetraformis TF071, while clade 2 contained *Defluviicoccus* GAO-HK, as expected, together with *Defluviicoccus* SSA4, and two recovered genomes from the treatment plant at Fredericia Jutland in Denmark (FRED MAXAC 307 and 378). The sequence from *Ca.* D seviourii located within the Clade III, as expected (McIlroy et al., 2010), while the bin129 genome sequence obtained from the marine habitat, and attributed to *Defluviicoccus* (Slaby et al., 2017) and the sequence of the other Danish bacteria population from the Kalundborg and Sjaelland (KALU MAXAC 148) wastewater treatment plants appeared in separate clades, and quite distinct from pre-existing clades members I–IV (Figure 3). Thus, we propose here the formation of a new clade (V) to accommodate the KALU MAXAC 148 population. The systematics of the source organism of the bin129 genome will be addressed later.

Average nucleotide identity (ANI) comparisons between all pairs of the nine *Defluviicoccus* genomes (Table 1) were within the range: 76–80%, consistent with each being, at minimum, a distinct species, according to the interpretation of Jain et al. (2018).

**FIGURE 4:**
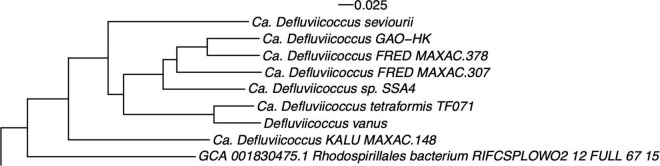
Whole genome phylogenetic structure of members of the genus *Defluviicoccus*, based on genomes listed in Table 1 and closely related draft and reference genomes. The *Defluviicoccus* specific cluster is shown here and the full tree is provided as Supplementary Figure 3. Note that the Slaby bin 129 genomes assumed a distal position with respect to this region and is not shown (see Section “Methods: Comparative Analysis of *Defluviicoccus* Genomes”).

A whole genome phylogenetic analysis (Figure 4 and Supplementary Figure 3), while having fewer and more sparsely populated clusters, reflects closely the relationships revealed by the 16S rRNA gene sequence analyses. Thus, the *D. vanus^T^* genome sequence appears in the same clade as that of *Ca.* D. tetraformis TF701, and those from the populations in Hong Kong (GAO-HK), Singapore (SSA4), and the Fredericia plant in Denmark (FRED MAXAC 307 and 378), all clustered in the same clade. The *Ca*. D. seviourii genome was adjacent to these six genomes, and that from the Kalundborg WWTP, Sjaelland Danish plant (KALU MAXAC 148) was adjacent to the other seven genomes above. The bin 129 genome sequence reported by Slaby et al. (2017) was distinct from all these *Defluviicoccu*s sequences, assuming a distant position to other members in the tree (Supplementary Figure 3).

### Are Members of *Defluviicoccus* Misplaced in the Family *Rhodospirillaceae* or Should They Be Relocated in the *Geminococceae* as Proposed by Hördt et al. (2020)?

Based on Genome Taxonomy Database (GTDB) systematics of the *Ca.* D. seviourii extant genome, Onetto et al. (2019) suggested that *Defluviicoccus* should be removed from the family *Rhodospirillaceae* and instead reclassified into a candidate family 2-12-FULL-67-15. Furthermore, in a recent reorganization of the *Alphaproteobacteria*, based on 16S rRNA gene sequences, it was proposed that members of the genus *Defluviicoccus* should be placed within the family *Geminicoccaceae*, with the caveat that *Defluviicoccus* may be assigned to its own family if sufficient evidence became available from additional whole genome data (Hördt et al., 2020).

**FIGURE 5:**
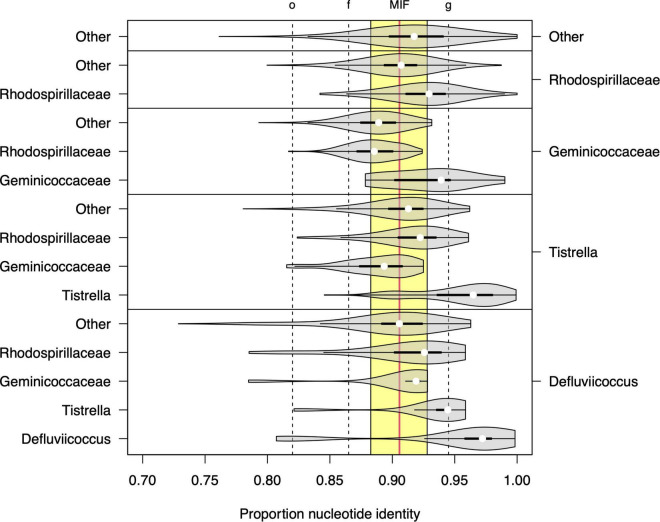
Sequence identity statistics of full length 16S SSU-rRNA sequences among members of genera *Defluviicoccus* and *Tistrella*, the families *Geminicoccaceae* and *Rhodospirillaceae*, and other members of the order *Rhodospirillales*. Each violin plot shows the distribution of the proportion of nucleotide identity (PNI) between members of two microbial groups (listed on the right and left axes, respectively). The vertical dashed lines show the values of the minimal inclusion thresholds for different taxonomic levels (g, genus; f, family; o, order) defined by Yarza et al. (2014) and the yellow region delineates the range of mean PNI values from intra-family groups within order *Rhodospirillales*, with the overall mean shown as vertical solid red line (denoted as MIF: median inter-family).

The 16S rRNA sequence analysis was expanded here to examine more closely the proposals by Hördt et al. (2020), where *Defluviicoccus* and *Tistrella* were considered for placement within a single family, the *Geminicoccaceae* (order *Rhodospirillales*), rather than their existing location within the family *Rhodospirillaceae* (Figure 5). Direct comparison of per-group proportion nucleotide identity (PNI) statistics (Figure 5) showed that the median differences in PNI between both *Defluviicoccus* and *Tistrella* and *Geminicoccaceae* were larger than those observed between both *Defluviicoccus* and *Tistrella* and the *Rhodospirillaceae*, with the former distances being larger than the median of the inter-family distances observed within members of the *Rhodospirillales.* However, the majority are still above the conservative lower threshold for inter-family variation as defined by Yarza et al. (2014). The differences between *Defluviicoccu*s and *Tistrella* are consistent with these two genera being related at either intra-family or intra-order level. Therefore, while it seems clear that sequences from members of the genus *Defluviicoccus* and those from members of the genus *Tistrella* show a magnitude of difference inconsistent with them both belonging to the family *Geminicoccaceae*, as proposed by Hördt et al. (2020), these findings support the view that members of the genus *Defluviicoccus* are best considered as a separate family within the order *Rhodospirillales*.

This situation highlights that much is still to be learned about *Defluviicoccus* systematics, shown by a recent global survey of activated sludge plants of many different configurations (Dueholm et al., 2021) and based on 16S rRNA sequence data to consist of at least 18 putative new species. What is not yet clear is whether they all belong to the currently existing five clades, or additional clades are required to accommodate them.

### General Features of the *Defluviicoccus* Genomic Sequence Data

The genomic data reveal potential aspects of behavior of these organisms not exposed by experimental data, suggesting that some of these genes may not be expressed in wastewater treatment plants, at least. Thus, all the *Defluviicoccus* genomes, with one exception (genome bin 129), but including the filamentous *Ca.* D. seviourii, contained genes encoding the production of components of bacterial flagellar synthesis (Supplementary Data File 1). This is despite *Defluviicoccus* never having been seen to show motility or to possess such organelles. They also possess several genes encoding proteins dedicated to chemotaxis (Supplementary Data File 1), although not in the genomes of *D. vanus^T^*, *Defluviicoccus* clone SS4, *Ca*. D. seviourii clone, and genome bin129.

Equally interesting is that genomes of all except *Defluviicoccus* bin 129 possess the *kaiC* gene, and, except in the case of the KALU MAXAC 148 genome, the *kaiB* gene, which encode circadian clock-related proteins (Dvornyk et al., 2003). Furthermore, some have the genes associated with conjugation. Thus, *D. vanus^T^*, *Ca.* D. tetraformis, *Defluviicoccus* GAO-HK, and FRED MAXAC 307 and 378 have the conjugal transfer protein encoding gene *traG*, while *Defluviicoccus* SSA4, *Ca*. D. seviourii, genome bin 129, and MAXAC 148 possess the genes encoding the pilus assembly protein *cpfA* and the conjugal transfer protein *cpaF.* The bin 129 genome alone has the gene for the conjugal mating pair stabilization protein *traN* (Supplementary Data File 1). With the exception of *D. vanus^T^, Defluviicoccus* GAO-HK, and *Defluviicoccus* strains MAXAC 307 and 378, they also possess the genes for complete or partial synthesis of pili.

### Genes Involved in Substrate Uptake in *Defluviicoccus*

The *D. vanus^T^* genome contains the genes *acsA* (acetyl-CoA synthetase, EC6.2.1.1), *ackA* (acetate kinase, EC 2.7.2.1), and *pta* (phosphate acetyl/butyryltransferase, EC2.3.1.8), allowing assimilation and activation of acetate and involving the cation/acetate symporter *actP* (Table 2). These were also present in all but the bin129 genome, which lacked genes *ackA* and *pta*, although their copy numbers varied markedly in the remaining members (Supplementary Data File 1). Genes encoding acetyl CoA synthetase were also present in all *Defluviicoccus* genomes in multiple copies (Table 2 and Supplementary Data File 1). Additionally, all *Defluviicoccu*s genomes, except KALU MAXAC 148 and that of bin129 genome, contained a gene encoding the succinate-acetate/proton symporter *satP* (Sun et al., 2018).

**TABLE 2:** Selected genes related to substrate and amino acid uptake present in genomes from members of the genus *Defluviicoccus.*

Gene symbol	Description	Presence/absence within genome								
		*D. vanus*	*Ca. D. tetra. TF071*	*Ca. D.* sp. *SSA4*	*Ca. D.* sp. *Fred MAXAC 307*	*Ca. D.* sp. *Fred MAXAC 378*	*Ca. D.* sp. *GAO-HK*	*Ca. D. seviourii*	*Ca. D* sp. *Kalu MAXAC 148*	*Ca. D.* sp. *bin 129*
*actP*	Cation/acetate symporter	+	+	+	+	+	+	+	+	+
*ackA*	Acetate kinase	+	+	+	+	+	+	+	+	−
*acsA*	Acetyl-coenzyme A synthetase	+	+	+	+	+	+	+	+	+
*pta*	Phosphate acetyltransferase	+	+	+	+	+	+	+	+	−
*satP*	Succinate-acetate/proton symporter	+	+	+	+	+	+	+	−	−
*yhdW*	Putative amino-acid ABC transporter-binding protein	+	+	+	+	−	+	+	+	+
*yhdY*	Putative amino-acid ABC transporter-binding protein	+	+	+	+	−	+	+	+	+
*putP*	Sodium/proline symporter	+	+	+	+	+	+	+	+	−
*gltS*	Sodium/glutamate symporter	+	+	+	+	+	+	+	+	−
*sglT*	Sodium/glucose cotransporter	+	+	+	+	+	+	+	−	−
*glnM*	Putative glutamine ABC transporter permease protein	−	−	+	−	−	−	−	+	+
*glnH*	ABC transporter glutamine-binding protein	−	−	+	−	+	−	−	−	+
*ABC.PA.S*	Polar amino acid transporter, substrate-binding protein	+	+	+	+	+	+	+	+	+
*gntT*	H+/gluconate symporter and related permeases	+	−	+	+	+	+	−	−	−
*xylE*	MFS transporter, SP family, sugar: H+ symporter	+	+	+	+	−	+	−	−	−
*gltP*	Proton/glutamate-aspartate symporter	−	−	−	−	−	+	−	−	−
*fucP*	L-fucose-proton symporter	−	−	−	−	−	−	−	+	−
*frcA*	Fructose import ATP-binding protein FrcA	−	−	−	−	−	−	−	+	−
*fruA*	Fructose PTS system EIIBC or EIIC component	+	+	−	+	−	+	−	−	−
*manX*	Mannose PTS system EIIA component	+	+	+	+	+	+	+	+	+
*mglA*	Galactose/galactoside import ATP-binding protein	−	−	−	−	−	−	−	−	+
*urtABCDE^a^*	Urea transport system ATP-binding proteins	+	+	+	+	+	+	+	+	+
*amt*	Ammonium transporter, Amt family	+	+	+	+	+	+	+	+	+

*^a^Largely intact across genomes but refer Supplementary Data File 1 for details of missing components.*

All *Defluviicoccus* genomes except bin129 contain the genes encoding a propionyl CoA synthetase, responsible for activation of propionate and a propionyl CoA carboxylase, which converts propionyl CoA to methyl malonyl CoA. However, no corresponding genes encoding the cation/propionate symporter (*pctP*) were identified, and while the succinate-acetate/proton symporter, *satP* was observed in some genomes, there is no direct evidence that it can transport propionate (Sun et al., 2018). Thus, all genomes have dedicated genes for uptake and subsequent metabolism of acetate but not for propionate (Supplementary Table 1), which seems to suggest, as Burow et al. (2008a) did, that acetate and propionate share the same transporter/s in *Defluviicoccus*. Its members appear to assimilate propionate at a higher rate than acetate (Oehmen et al., 2005a; Dai et al., 2007), and is the preferred substrate when both are available. However, although *Ca.* Competibacter has a negligible rate for assimilation of propionate, its assimilation by Ca. Accumulibacter is faster than *Defluviicoccus* (Oehmen et al., 2005a,b), suggesting different *K*_m_ values for the two substrates. Furthermore, unlike *Defluviicoccus*, there was no delay in their assimilation rates when supplied alternatively in the feed (Lu et al., 2006), which has led to the suggestion (Oehmen et al., 2005a,^2006^) that regularly alternating the feed substrate would provide an elegant strategy for controlling the abundances of both *Ca.* Competibacter and *Defluviicoccus* GAO. However, its feasibility for use in full-scale EBPR plants has received little interest.

Whether *Defluviicoccus* can assimilate butyrate under anaerobic conditions is still the subject of controversy, and the scant literature available suggests that not all members have this ability (Burow et al., 2008a; He and McMahon, 2011; Begum and Batista, 2014; Izadi et al., 2020). This character may be one of many that are not present universally in all *Defluviicoccus* populations. Certainly, the enzymes potentially involved in butyrate metabolism were not distributed equally among these genomes (Supplementary Data File 1). Thus, genome bin 129 alone encoded genes (*bcd*; EC1.3.8.1) for butyryl CoA dehydrogenase. Only *Ca*. D. tetraformis lacked that encoding 3-hydroxybutyryl CoA dehydrogenase (EC 1.1.1.157; K00074 synonyms *paaH*, *hbd*, *fadB*, and *mmgB*) enabling the remainder to convert 3-hydroxybutyrate to butyryl-CoA. Genes encoding isobutyryl-CoA mutase (EC 5.4.99.13; K11942, *icmF*) were found in all genomes except *Ca. Defluviicoccus* HK-GAO, *Ca*. D. tetraformis, and genome bin 129, while 4-hydroxybutyryl dehydratase (EC 4.2.1.120; K14534, *abfD*) was encoded in all genomes except *Defluviicoccus* MAXAC 307 and 378, and the genome bin 129. No genes encoding butyryl-CoA synthetase (EC 6.2.1.2) were found in any of these genomes, suggesting that growth on butyrate is unlikely for the strains examined here, although whether the same system/s used to transport and activate acetate and propionate are used for butyrate remains unresolved by this analysis. In *Escherichia coli*, the *satP* transporter discussed above has been shown to have co-selectivity for butyrate (Sun et al., 2018), although no related data yet exist for *D. vanus^T^*. Wang et al. (2021a) showed that at high temperatures, where *Defluviicoccus* was thought to have a competitive advantage over A*ccumulibacter* PAO (Lopez-Vazquez et al., 2009), butyrate impacted negatively on *Defluviicoccus* abundance. More recently, however, Wang et al. (2021b) have shown that at these high temperatures, highly enriched cultures of *D. vanus^T^* assimilated both butyrate and isobutyrate, albeit at much slower rates of assimilation than either acetate and propionate, and uncommon mid-chain (6–14 carbons) poly-β-hydroxyalkanoates, polyhydroxy-4-methylvalerate (PH4MV), polyhydroxyhexanoate (PHH_X_), and polyhydroxy-2-methyl hexanoate (H2MH_X_) were synthesized by them anaerobically.

The genome sequence data presented here suggest that *D. vanus^T^* and all the other *Defluviicoccus* genomes contain genes encoding diverse ABC amino acid transporters (Supplementary Data File 1), and with the exception of *Defluviicoccus* FRED MAXAC 378, the *aap* systems for transporting L-amino acids, namely, *yhdW* and *yhdY* (Table 2), permitting them to assimilate a wide range of amino acids, despite not always being implicated in earlier FISH/MAR data (Burow et al., 2008a). Not all the encoding genes for these transport systems were present universally in these genomes, and so while all possessed the genes (*liv* family) for transport of branched amino acids (Supplementary Data File 1), those encoding the Na^+^-linked symporters, namely, Na^+^/proline (*putP;* K03307), Na^+^/glucose (*sglT*; K03307), and Na^+^/glutamate (*gltS*; K03312) symporters, were present only in *D. vanus^T^*, *Defluviicoccus* GAO-HK, *Ca.* D. seviourii (consistent with the analysis of Onetto et al., 2019), and *Ca.* D. tetraformis (Table 2). Equally, individual copy numbers of most of these genes varied with the strain (Supplementary Data File 1). Polar amino acid dedicated transporter genes (*glnM, COG0765*, and *K09970*) were found in *Defluviicoccus* SS4, genome bin129, and KALU MAXAC 148, while *glnH* (COG0834) was found in *Defluviicoccus* SS4, genome bin 129, and FRED MAXAC 378. Substrate binding polar amino acids (ABC.PA.S; COG0834, K02030) were present in all *Defluviicoccus* genomes (Table 2).

Considerable disagreement exists in the literature as to whether all *Defluviicoccus* can grow anaerobically on glucose or other sugars as sole carbon sources, although FISH/MAR has shown that *Ca*. Competibacter denitrificans can assimilate glucose under both aerobic and anaerobic conditions (McIlroy et al., 2014). Maszenan et al. (2005) managed to culture *D. vanus^T^* with glucose aerobically as sole carbon source, but it grew very slowly. Wong and Liu (2007) have shown with FISH/MAR that *Defluviicoccus-*related tetrads in a laboratory reactor could utilize acetate, lactate, propionate, and pyruvate, but not aspartic acid and glucose into PHA under anaerobic condition, yet when *D. vanus^T^* was cultured in continuous anaerobic–aerobic conditions, it took up glucose with concurrent glycogen consumption and PHA production, the assumption being that it was the same strain.

Genomic analyses revealed the presence of a gene encoding a fructose import ATP binding protein in *Defluviicoccu*s KALU MAXAC 148 (*frcA;* K10554), a fructose PTS system (*ELLBC*/fruA, EC:2.7.1.202) in *D. vanus^T^, Ca*. D *tetraformis*, *Defluviicoccus* GAO-HK, and *Defluviicoccus* FRED MAXAC 307, while the mannose PTS system (*ELLA* component, *manX*, EC:2.7.1.191) was present in all genomes. A galactose/galactoside import encoding ATP protein (*mglA*) was seen only in the genome bin129.

Genomes of *D. vanus^T^, Ca*. D. tetraformis, *Defluviicoccus* sp. SSA4, and *Defluviicoccus* FRED MAXAC 378 each possessed a single gene copy encoding a glucose/mannose co-transporter (*glcP*, Table 2). It was not identified in the genomes of *Ca. Defluviicoccus* GAO-HK, *Ca.* D. seviourii, or *Defluviicoccus* KALU MAXAC 148. Given that both *Defluviicoccus* GAO-HK and *Defluviicoccus* KALU MAXAC 148 possessed one or more copies of a sodium/glucose symporter gene (*sglT*), it seems probable that potentially these strains may assimilate glucose, while those with neither gene, i.e., *Defluviicoccus* FRED MAXAC 307, nor the genome bin29 may be unable to.

All possessed transporter encoding often several copies of the genes for urea (*urt* family) and ammonium (*amt* family) transport, although a sulfate transporter gene was found only in the genomes of *D. vanus^T^*, *Ca*. D tetraformis, and *Defluviicoccus* KALU MAXAC 148 and FRED MAXAC 378 (Supplementary Data File 1).

### Mechanisms of Anaerobic and Aerobic Substrate Uptake in *Defluviicoccus* Strains

Active transport processes are required in *Defluviicoccus* for anaerobic acetate and propionate membrane transport, where acetate and propionate probably share the same transporter. Burow et al. (2008a,b) used metabolic inhibitors in attempts to resolve details of the transport mechanisms with a highly enriched culture of clade 1 *Defluviicoccus*. While inhibitor selectivity is always a concern with such approaches, their data were consistent with anaerobic acetate assimilation depending on both a proton motive force (pmf) and a Na^+^ potential, with the former being the main contributor. They suggested that ATPases and P efflux through the membrane (involving the *pit* transporter) played no role in pmf establishment and thus was quite different to the situation in *Ca*. Accumulibacter PAO and *Ca.* Competibacter (Saunders et al., 2007; Seviour and McIlroy, 2008; Oehmen et al., 2010). Instead, the pmf was more likely established by H^+^ efflux coupled to electron transport-linked reduction of fumarate in the reductive branch of the TCA cycle (see below). Genes encoding fumarate reductases were present in all the *Defluviicoccus* genomic sequences examined here, including *D. vanus*^T^** (Supplementary Data File 1).

Burow et al. (2008a,b) also suggested that the anaerobic Na^+^ motive force (smf) could be generated by extrusion of a methylmalonyl-CoA decarboxylation-linked efflux of Na^+^ across the cell membrane. Methylmalonyl-CoA is an intermediate in the succinate–propionate pathway, with the encoding genes for methylmalonyl-CoA decarboxylase present in all except the bin 129 genome, and leading to the formation of 3-hydroxy valeryl-CoA (Seviour and McIlroy, 2008). The key enzyme in this pathway, methylmalonyl-CoA mutase, converts methylmalonyl-CoA to propionyl-CoA, and genes encoding it were detected in all the *Defluviicoccus* genomes, with the exception of genome bin 129. Again, neither process is found in *Ca*. Accumulibacter, although a fumarate reductase generated membrane pmf potential was reported in *Ca.* Competibacter (Saunders et al., 2007; McMahon et al., 2010). The same system probably exists in those *Defluviicoccus* strains possessing the genes encoding for methylmalonyl-CoA decarboxylase. Although this enzyme is absent from both genome bin 129 and *Defluviicoccus* KALU MAXAC 148 genomes (Supplementary Data File 1), a fumarate reductase-generated pmf may well be used instead.

Under aerobic conditions, *Ca*. Accumulibacter can assimilate P by a process driven by a pmf established by P efflux of P_i_ across the membrane (Saunders et al., 2007; McIlroy et al., 2014) involving the low-affinity *pit*, and operating when P_i_ is plentiful. A high-affinity *pst* system operates at lower Pi levels, or possibly both are used simultaneously (Burow et al., 2008a). It has long been thought (Seviour and McIlroy, 2008; McMahon et al., 2010) that possession of a *pit* gene distinguished all PAO from the GAO, in that *pit* appeared to be absent from *Ca*. Competibacter (McIlroy et al., 2014) and some earlier published *Defluviicoccus* genomes (Nobu et al., 2014; Onetto et al., 2019), Furthermore, while the *pst* gene occurred in all genomes examined here, often in multiple copies (Supplementary Data File 1), the *pit* gene was also detected, but only in *Defluviicoccus* FRED MAXAC 307, the genome bin 129 genomes, and in *Defluviicoccus* GAO-HK, as reported previously by Wang et al. (2014). No GAO can store polyphosphate under the aerobic conditions tested to date. Whether these *pit* genes are homologous to the *pit* in the PAO, or whether they are expressed are not known. What seems clear is that much remains to be clarified about regulation of P metabolism in *Defluviicoccus* and the other GAO.

### Source of Reducing Power and Energy for Polyhydroxyalkanoate Synthesis by *Defluviicoccus* Under Anaerobic Conditions

This aspect of GAO molecular physiology has been controversial, as it has been for *Ca*. *Accumulibacter* PAO (Oehmen et al., 2007; Burow et al., 2009; Zhou et al., 2010). It is likely to differ fundamentally from the situation in PAO since *Defluviicoccus* possess glycogen and not polyphosphate as their major stored energy source. Equally controversial is how they balance their intracellular redox under anaerobic conditions. Experimental and genomic data suggest that *Ca*. Accumulibacter can operate the TCA cycle anaerobically to generate energy in addition to that arising from polyphosphate hydrolysis, and reducing power, since it possesses an unusual cytochrome b/b_6_, which allows succinate dehydrogenase to function in the forward direction to produce fumarate (García Martín et al., 2006; Skennerton et al., 2015). This transformation step is not possible in *Defluviicoccus.* Debate has focused on whether these GAO obtain all or only some of their energy and reducing power for PHA synthesis from anaerobic glycogen catabolism *via* the Embden Meyerhoff Parnas pathway (Burow et al., 2009; Zhou et al., 2010). What now seems more likely is that *Defluviicoccus* can use the reductive branch of the TCA cycle to balance its intracellular redox. Key features involve fumarate reductase, which converts fumarate to succinate and the associated succinate–propionate pathway leading *via* methylmalonyl-CoA decarboxylase and methylmalonyl-CoA to PHA storage products. While PHA production from an acetate feed in *Ca.* Accumulibacter is 3-polyhydroxybutyrate (PHB) only, *Defluviicoccus* produces both 3-polyhydroxyvalerate (PHV, 25%) and 3-polyhydroxybutyrate (73%) (Zhou et al., 2008). With propionate as substrate, an increased production of propionyl CoA (Oehmen et al., 2007) and the subsequent operation of the succinate–propionate pathway supplying precursors for the methylmalonyl-CoA pathway lead to the synthesis of 3-polyhydroxyvalerate and 3-hydroxy 2 methylvalerate. As *Ca.* Accumulibacter lacks the methylmalonyl-CoA pathway (Oehmen et al., 2007, 2010), no 3-hydroxy 2 methylvalerate is produced (Seviour and McIlroy, 2008). The genes encoding fumarate reductase, propionyl-CoA-carboxylase, and both methylmalonyl-CoA-decarboxylase, converting methylmalonyl-CoA to propionyl-CoA, and its mutase, converting succinyl-CoA to methylmalonyl-CoA, are found in most of the *Defluviicoccus* genomes examined here, including *D. vanus*^T^ (Supplementary Data File 1).

### Central Carbon Metabolism in the *D vanus*^T^ Genome; How Do They Compare to Other Members of the Genus *Defluviicoccus?*

The *D. vanus*^T^ genome encodes a complete Emden–Meyerhoff pathway (EMP), tricarboxylic cycle (TCA), gluconeogenesis pathway, and a complete module for pyruvate oxidation to acetyl-CoA (Table 3). These pathways are largely recapitulated across other genomes in members of the genus *Defluviicoccus*, albeit with missing components, notably in *Defluviicoccus* FRED MAXAC 378, Defluviicoccus GAO_HK, and the genome bin 129. Such gaps most probably are consequences of fractionated genome assemblies in the case of those constructed from short read data, and instances of reduced gene sequence quality with those constructed with long read data. In contrast to these pathways, the pentose phosphate pathway, the Enter–Doudoroff pathway, and the photorespiration pathway all had substantive numbers of missing enzymes and appear to be non-functional (Table 3).

**TABLE 3:** Summary of KEGG modules detected in genomes of members of genus *Defluviicoccus.*

Module*^a^*	Description	Status*^b^* | Number of genes detected in KEGG module								
		*D. vanus*	*Ca. D. tetra-formis TF071*	*Ca. D.* sp. *SSA4*	*Ca. D.* sp. *Fred MAXAC 307*	*Ca. D.* sp. *Fred MAXAC 378*	*Ca. D.* sp. *GAO-HK*	*Ca. D. seviourii*	*Ca. D* sp. *Kalu MAXAC 148*	*Ca. D.* sp. *Bin 129*
**Carbohydrate metabolism (central)**										
M00001	Glycolysis, glucose ⇒ pyruvate (EMP)	C | 12	C | 13	C | 11	+ | 10	+ | 9	? | 9	C | 12	C | 13	? | 7
M00003	Gluconeogenesis, oxaloacetate ⇒ fructose-6P	C | 9	C | 10	C | 9	+ | 8	? | 6	? | 7	+ | 9	+ | 8	+ | 7
M00307	Pyruvate oxidation, pyruvate ⇒ acetyl-CoA	C | 6	C | 5	C | 5	C | 5	C | 5	C | 5	C | 5	C | 6	C | 4
M00009	Citrate cycle (TCA cycle, Krebs cycle)	C | 20	C | 21	C | 21	C | 21	C | 20	C | 21	C | 21	+ | 21	+ | 14
M00007	Pentose phosphate pathway, non-oxidative	+ | 3	+ | 3	+ | 3	+ | 3	? | 2	+ | 3	+ | 3	+ | 3	+ | 3
M00308	Semi-phosphorylative Entner–Doudoroff	? | 2	? | 2	? | 2	? | 1	? | 2	? | 3	? | 2	+ | 4	+ | 4
M00552	D-galactonate degradation, De Ley–Doudoroff	? | 2	? | 2	? | 2	? | 1	? | 1	? | 2	? | 2	+ | 4	? | 2
M00854	Glycogen biosynthesis, glucose-1P ⇒ glycogen	C | 5	C | 5	C | 5	C | 5	C | 5	C | 5	C | 5	C | 4	− | 0
M00855	Glycogen degradation, glycogen ⇒ glucose-6P	**C | 5**	C | 4	C | 4	+ | 4	+ | 4	+ | 4	+ | 4	+ | 4	− | 0
M00565	Trehalose biosynthesis	C | 7	C | 7	+ | 6	C | 7	+ | 6	C | 7	C | 7	C | 6	− | 0
M00012	Glyoxylate cycle	− | 4	− | 4	− | 4	− | 4	− | 4	− | 4	− | 3	− | 4	+ | 4
M00373	Ethylmalonyl-CoA pathway	C | 13	C | 13	C | 13	C | 13	? | 10	C | 13	+ | 12	+ | 12	? | 6
M00532	Photorespiration	? | 5	? | 5	? | 5	? | 5	? | 4	? | 5	? | 5	? | 5	? | 4
M00741	Propanoyl-CoA metabolism	C | 4	C | 4	C | 4	C | 4	C | 4	C | 4	+ | 3	+ | 3	? | 1
**Energy metabolism (Carbon fixation)**										
M00173	Reductive citrate cycle (Arnon–Buchanan cycle)	? | 18	+ | 20	? | 18	+ | 19	+ | 19	+ | 19	? | 19	? | 19	? | 13
**Energy metabolism (Nitrogen)**										
M00175	Nitrogen fixation, N2 ⇒ NH3	C | 4	C | 4	C | 3	C | 3	C | 3	C | 4	C | 3	C | 3	− | 0
M00531	Assimilatory nitrate reduction, NO3 ⇒ NH3	− | 0	C | 2	− | 0	C | 2	C | 2	− | 0	C | 2	− | 0	− | 0
M00530	Dissimilatory nitrate reduction, NO3 ⇒ NH3	− | 0	? | 1	− | 0	? | 1	+ | 4	− | 0	? | 1	− | 0	+ | 4
M00529	Denitrification, NO3 ⇒ N2	? | 1	? | 1	? | 1	? | 1	? | 4	? | 2	− | 0	? | 1	? | 4
**Energy metabolism (Sulfur)**										
M00176	Assimilatory sulfate reduction	? | 3	C | 5	? | 3	C | 5	C | 5	+ | 4	C | 5	+ | 4	? | 2
M00596	Dissimilatory sulfate reduction	− | 0	− | 0	− | 0	− | 0	− | 0	− | 0	− | 0	− | 0	+ | 3

*^a^KEGG Module: canonical modules can be examined via the URL: https://www.genome.jp/kegg-bin/show_module?M<NNNNN>.*

*^b^Status: inferred from BlastKOALA Pathway Reconstruction Results as follows: “C”: a complete pathway, either defined as such by BlastKOALA or confirmed by manual review of multiple sources of annotation [in bold]; “+”, pathways that are largely intact (no more than 2 missing blocks as defined by BlastKOALA); “?”, pathways that are classified as BlastKOALA as incomplete or are missing more than 2 blocks; “–”, pathways that are classified as BlastKOALA as absent.*

The glyoxylate shunt pathway (KEGG M00012) represents an important mechanism for the conversion of acetyl-CoA into both gluconeogenic and anaplerotic precursors (Ensign, 2006; Renilla et al., 2012), and was classified by BlastKOALA here as only partially complete in all *Defluviicoccus* genomes, including *D*. *vanus*^T^. With the exception of the genome bin 129, this arose from the absence of the gene encoding isocitrate lyase (*aceA*; KEGG orthology K01637, EC: 4.1.3.1; also not annotated by Prokka), which is a diagnostic enzyme for this pathway (Supplementary Data File 1). Furthermore, *Ca.* D. *seviourii* lacked the gene encoding malate synthase (KEGG Orthology K01638 and EC: 2.3.3.9), as noted previously (Onetto et al., 2019; Supplementary Data File 1). Because all other gene-encoded enzymes of the pathway are also members of the TCA cycle, it was therefore considered to be absent in all genomes, except genome bin 129, where it was also classified as incomplete in missing the *mdh* gene (malate dehydrogenase; K00024 and EC:1.1.1.37) (Table 3 and Supplementary Data File 1). The presence of this pathway has also been revealed for *Ca.* Accumulibacter (Burow et al., 2009) based on targeted enzyme inhibitor studies and genome sequence data for *Ca.* C. denitrificans (McIlroy et al., 2014).

In the absence of the glyoxylate shunt pathway, the ethylmalonyl-CoA pathway (KEGG M00373) is thought to provide an alternative mechanism for conversion of C2 compounds, notably acetyl-CoA (Alber, 2011; Anthony, 2011; Schada von Borzyskowski et al., 2020) to the intermediates malate and succinyl-CoA in the TCA cycle. As annotated by BlastKOALA, the gene encoding (3S)-malyl-CoA thioesterase (*mcl2*; K14451, EC:3.1.2.30), which converts (3S)-malyl-CoA to malate, was a false negative and its presence was confirmed subsequently in all *Defluviicoccus* genomes by examining annotations from Prokka (in *D. vanus^T^* see gene CDGBEKEE_03341; Supplementary Data File 1). Hence, in the case of *D. vanus^T^*, *Ca.* D. tetraformis TF071, *Defluviicoccus* SSA4, *Defluviicoccus* FRED MAXAC 307, and *Defluviicoccus* GAO-HK, we conclude that the ethylmalonyl-CoA pathway is complete (Table 3 and Supplementary Data File 1). This pathway showed various degrees of incompleteness for the remaining genomes. Hence, the *Defluviicoccus* FRED MAXAC 378 genome was missing the key genes encoding crotonyl-CoA reductase (*ccr;* K14446), (2R), and ethylmalonyl-CoA mutase, the diagnostic gene for this pathway (*ecm;* K14447), and (2S) methyl succinyl-CoA dehydrogenase (*mcd* K14448), while both *Defluviicoccus* KALU MAXAC 148 and *Ca.* D. seviourii genomes lacked the key gene encoding methylmalonyl-CoA/ethylmalonyl-CoA epimerase (*epi* K05606, EC:5.1.99.1). Thus, either these genes are redundant, the ethylmalonyl-Co-A pathway cannot function in these strains, or they are false negatives as a result of draft genome incompleteness.

### Comparative Genomics of Glycogen and Trehalose Metabolism Within Genus *Defluviicoccus*

**FIGURE 6:**
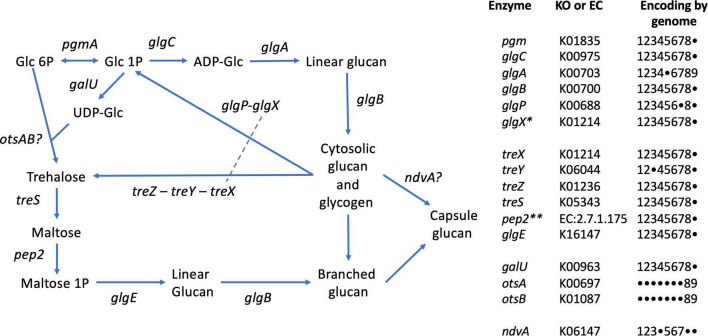
Glycogen and trehalose biosynthetic pathways conserved in members of the genus *Defluviicoccus*, based on Figure 1 of Chandra et al. (2011). The tabulation on the right lists the enzymes and presence of encoding genes in the 9 genomes analyzed in this study, using the following numeric key: 1: *Defluviicoccus vanus*; 2: *Ca.* Defluviicoccus tetraformis TF071; 3: *Ca.* Defluviicoccus. sp. SSA4; 4: *Ca.* Defluviicoccus Fred MAXAC 307; 5: *Ca.* Defluviicoccus Fred MAXAC 378; 6: *Defluviicoccus* GAO-HK; 7: *Ca.* D. seviourii; 8: *Ca*. Defluviicoccus Kalu MAXAC 148; 9: *Defluviicoccu*s bin129. A closed circle • denotes genes not identified in the corresponding genome. Full enzyme names as follows: *pgm*, phosphoglucomutase; *glgC*, glucose-1-phosphate adenylyltransferase; *glgA*, glycogen synthase; *glgB*, 1,4-alpha-glucan branching enzyme; *glgP*, glycogen phosphorylase; *glgX*, glycogen debranching enzyme; *treX*, maltooligosyltrehalose synthase; *treY*, maltooligosyl trehalose synthase; *treZ*, malto-oligosyltrehalose trehalohydrolase; *treS*, trehalose synthase/amylase; *pep2*, maltokinase; *glgE*, alpha-1,4-glucan:maltose-1- phosphate maltosyltransferase; *galU*, UTP-glucose-1-phosphate uridylyltransferase; *otsA*, trehalose-6-phosphate synthase; *otsB*, trehalose-phosphate phosphatase; *ndvA*, beta-(1– > 2)glucan export ATP-binding/permease protein. *This appears to be a single type of glycogen debranching enzyme in the genus *Defluviicoccus;* dashed line in pathway schematic. ^**^As described by Chandra et al. (2011), in all genomes except *D. vanus*, *pep2* (maltokinase) formed a single continuous sequence with *treS* and identity was confirmed by sequence alignments; see Supplementary Data File 4.

Glycogen recycling is a key feature of the GAO phenotype, expressed in the cyclical anaerobic–aerobic conditions operative in EBPR bioprocesses (Lv et al., 2014). Glycogen degradation is thought to be the primary source of reducing equivalents in *Defluviicoccus* for formation of intracellular PHA, synthesized from either carbon substrates assimilated in the anaerobic feed phase and/or from organic acids derived from catabolism of glycogen (Liu et al., 1997). As mentioned earlier, aerobic degradation of PHA in the aerobic phase provides substrates for glycogen synthesis through gluconeogenesis for which the complete pathway is present in *D. vanus*^T^ (see above). As annotated by BlastKOALA (Table 3 and Figure 6), and as expected, all other *Defluviicoccus* genomes with the exception of genome bin 129 contain a complete canonical pathway for glycogen synthesis (KEGG M00854). In the case of glycogen degradation, BlastKOALA appears to have misannotated the glycogen debranching enzyme (*glgX*) as a false negative. Its presence in all *Defluviicoccus* genomes was confirmed subsequently from examining the Prokka annotation (CDGBEKEE_01736 in *D*. *vanus*^T^; Supplementary Data File 1).

The presence of the trehalose biosynthesis pathway in *Ca.* D. seviourii was noted by Onetto et al. (2019) and is thought to provide flexibility in energy storage and protection against stress (Onetto et al., 2019). Evidence for trehalose acting as an osmolyte in *Ca.* Accumulibacter has been reported (de Graaff et al., 2021), providing tolerance for saline environments in this group. A similar function for trehalose may be served in *Defluviicoccus. D vanus*^T^ also contains the genes for the complete trehalose biosynthetic pathway as defined by KEGG (M00565; Table 3 and Figure 6), where the *treX* (glgX)-*treY-treZ* genes encode the conversion of glycogen to trehalose (de Graaff et al., 2021). This pathway is present in genomes of the other genus members (Table 3 and Figure 6).

An alternative pathway facilitating reconversion of trehalose to a branched glucan *via* the production of maltose (Chandra et al., 2011) and mediated by proteins encoded by *treS-pep2-glgE*/*glgE1-glgB* is also present in both *D. vanus*^T^ and all genomes studied here (Table 3 and Figure 6). This latter pathway may be involved in formation of its exocellular capsular material (Chandra et al., 2011). We observed the *ndvA* glucan export transporter in 6 of the 9 genomes (Figure 6), giving at least some insight into the possible mechanisms underpinning capsule formation.

In contrast, the genes encoding the classical *galU-OtsA-OtsB* trehalose biosynthetic pathway was only observed in the case of the Kalu MAXAC 148 genome (Figure 6). Furthermore, the Rv3032/Rv3031 non-classical pathway, present in *Mycobacterium tuberculosis* and associated with the production of specialized glucans (reviewed in Chandra et al., 2011), was not observed in any *Defluviicoccus* genome.

### Pathways Related to Polyhydroxyalkanoate Metabolism in Genus *Defluviicoccus*

As discussed above, PHA cycling, and its complementary relationships to glycogen cycling in EBPR plants also represent key components of the GAO phenotype. As shown by earlier genomic analyses of other members of the genus *Defluviicoccus* (Nobu et al., 2014; Wang et al., 2014; Onetto et al., 2019), the *D. vanus*^T^ genome contains the genes encoding synthesis of the monomers PHB and PHV from acyl-CoA compounds derived from acetate and propionate metabolism, respectively. These are namely *phaA* and *phaB* from acetyl-CoA, and *fabF* and *fabG* from propionyl-CoA, and most likely butyryl-Co-A (Brune and Schink, 1992), although as discussed above, the fate of butyrate in some members of this genus remains unclear.

*D. vanus^T^* also possesses the genes for pathways for alternate PHA-monomer synthesis, related to fatty acid metabolism. These are *fabG* and *phaJ* (see genes CDGBEKEE_02222 and CDGBEKEE_02420; Table 4 and Supplementary Data File 1) (Wang et al., 2014). Two PHA synthases, *phaC* and *phaE*, respectively, and co-located in the genome, were identified, together with the *phaZ* PHA depolymerase (KO05973) (Table 4). *D. vanus*^T^ also harbors genes encoding three *PaaU*-like proteins, capable of reducing acetoacetyl-CoA to (S)-3-HB-CoA, with the R-3-HB-CoA isomer synthesized by *phaB* (Sagong et al., 2018). Other PHA proteins identified in the *D. vanus*^T^ genome include a PhaR-like repressor protein, a putative esterase-type PHB depolymerase and three short phasin proteins that possibly play a role in regulation and formation of the PHA granules (Grage et al., 2009). As discussed previously, catabolic degradation of PHA most probably proceeds through conversion of 3-HB-CoA to acetoacetyl-CoA *via* crotonyl-CoA (Grage et al., 2009), and the acetyl-CoA can then enter central metabolism with glycogen formation occurring *via* the gluconeogenesis pathway, as discussed above.

**TABLE 4:** Selected genes related to polymeric metabolism in genomes from members of the genus *Defluviicoccus.*

Gene symbol	Description	Presence/absence status within genome								
		*D. vanus*	*Ca. D. tetra. TF071*	*Ca. D.* sp. *SSA4*	*Ca. D.* sp. *Fred MAXAC 307*	*Ca. D.* sp. *Fred MAXAC 378*	*Ca. D.* sp. *GAO-HK*	*Ca. D. seviourii*	*Ca. D* sp. *Kalu MAXAC 148*	*Ca. D.* sp. *bin129*
**Glycogen and alpha glucans**										
*glgC*	Glucose-1-phosphate adenylyltransferase	+	+	+	+	+	+	+	+	−
*glgA*	Glycogen synthase	+	+	+	+	+	+	+	+	−
*glgB*	1,4-alpha-glucan branching enzyme	+	+	+	+	+	+	+	+	−
*glgP*	Alpha-1,4 glucan phosphorylase	+	−	+	+	+	+	+	−	−
*glgX*	Glycogen debranching enzyme	+	+	+	+	+	+	+	+	−
*treS*	Trehalose synthase/amylase	+	+	+	+	+	+	+	+	−
*Mak*	Maltokinase	+	+	+	+	+	+	+	−	−
*glgE^a^*	Alpha-1,4-glucan:maltose-1-phosphate maltosyltransferase	+	+	+	+	+	+	+	+	−
*treY*	Maltooligosyl trehalose synthase	+	+	−	+	+	+	+	+	−
*treZ*	Malto-oligosyltrehalose trehalohydrolase	+	+	+	+	+	+	+	+	−
*glgM*	Alpha-maltose-1-phosphate synthase	+	+	+	+	+	+	+	+	−
**Polyhydroxyalkanoate related**										
*phaA*	Acetyl-CoA acetyltransferase	+	+	+	+	+	+	−	+	+
*phaC*	Poly(3-hydroxyalkanoate) polymerase subunit C	+	+	+	+	+	+	+	+	+
*phaE*	Poly(3-hydroxyalkanoate) polymerase subunit E	+	+	+	+	+	+	+	+	−
*phaJ*	(R)-specific enoyl-CoA hydratase	+	−	+	+	+	+	+	+	+
**Polyphosphate and inorganic phosphate transport**										
*ppk*	Polyphosphate kinase	+	+	+	+	+	+	+	+	+
*pap*	Polyphosphate: AMP phosphotransferase	−	−	−	−	−	−	−	−	−
*ppx*	Exopolyphosphatase	+	+	+	+	+	+	+	+	+
*pstABC*	Phosphate transport system	+	+	+	+	+	+	+	+^b^	+
*pstS*	Phosphate-binding protein	+	+	+	+	+	+	+	−	−
*pitA*	Low-affinity inorganic phosphate transporter	−	−	−	+	−	+	−	−	+

*^a^In all genomes, glgB and glgB1 were observed, except for Kalu in which glgB and glgB2 were found.*

*^b^pstA was not observed in the Ca. D sp. Kalu MAXAC 148 genome.*

### Pathways Involving Utilization of Phosphate, Nitrogen, and Sulfur

The *D. vanus*^T^ genome also contains genes essential for polyphosphate synthesis and degradation, as do all *Defluviicoccus* genomes described here (Table 4).

Although the genes encoding the metabolic pathways discussed above appear mainly to be conserved across the genomes studied here, some variations become evident when examining the metabolism of nitrogen and sulfur compounds. While nitrogen fixation (KEGG M00175) appeared universally present, with the *nifHDK* operon being present in all *Defluviicoccus* genomes (Supplementary Data File 1), in *D*. *vanus*^T^, *Ca.* D. tetraformis, and *Defluviicoccus* GAO_HK, a complete *vnfDGK* operon encoding an alternative nitrogenase (Supplementary Data File 1) was present. Of the genes in the canonical denitrification pathway (KEGG Module M00529), only those for the nitrate reductase (*nar*), nitrite reductase (*nir*), and nitric oxide reductase (*nor*) gene families were observed, either singly or in various combinations (Supplementary Figure 4), with the sole exception of *Ca.* D. seviourii, which contained no genes from this canonical pathway. No other genome encoded a full denitrification pathway (Table 3 and Supplementary Data File 1), in agreement with all earlier physiological data (e.g., Wang et al., 2014). In the bin 129 genome, a partial denitrification pathway was observed, composed of *narG*, *narI*, *nary*, and *nirK*. Multiple genes in the *nar* family, namely, *narG*, *narI*, and *narY*, along with *norB*, were present in the *Defluviicoccus* FRED MAXAC 378 genome. Across all nine genomes, the most commonly observed gene was *norB*, which was present in all genomes except those of bin 129 and *Defluviicoccus* KALU MAXAC 148, suggesting that NO detoxification may be a common capability in members of this genus. In addition to *norB*, the *Defluviicoccus* GAO_HK genome also contained the *norC* gene. In the case of *Defluviicoccus* KALU MAXAC 148, the only denitrification-related gene found was *nirK*.

Marked differences were seen with the genes encoding assimilatory nitrate reduction, where those for that pathway were present in *Ca* D. tetraformis, *Defluviicoccus* FRED MAXAC 307, and FRED MAXAC 378 as well as *Ca*. D. seviourii (Table 3).

No genome studied here encoded a dissimilatory sulfate reduction, unlike *Ca.* C. denitrificans (McIlroy et al., 2014) (Table 3), although variations in genes encoding the assimilatory sulfate reduction pathway were observed in these genomes (Table 3). Thus, only those of *Ca.* D tetraformis, *Ca.* D. seviourii, and *Defluviicoccus* FRED MAXAC 307 and FRED MAXAC 378 possessed the full gene complement. Collectively, these observations highlight the potential for varied niche flexibility among members of genus *Defluviicoccus*.

## Conclusion

This paper reports the recovery of the complete, closed chromosomal genome of the GAO *Defluviicoccus* vanus^T^ taking advantage of new long read DNA sequencing technology. This approach has also facilitated recovery of a complete, closed genome of a conjugative plasmid, which can be challenging otherwise to reconstruct from short read DNA sequencing data alone (Arredondo-Alonso et al., 2017). We have provided here a complete set of gene annotations for all the currently available *Defluviicoccus* genomes in a form that can be used for systematic comparative analysis and genomic mining (Supplementary Data File 1). Both the raw sequencing data and the assembled genome sequences are publicly available from NCBI.

Phylogenomic functional comparisons in combination with recent whole genome sequences from other members of genus *Defluviicoccus* (Nobu et al., 2014; Wang et al., 2014; Onetto et al., 2019) were then conducted. The 16S rRNA gene and whole genome sequence comparisons give outcomes consistent with the previously proposed clade structures of members of this genus, as well as also suggesting the presence of at least one previously unrecognized clade/cluster, which we denote here as clade V (Figure 4). The proposed new clade is most evident in the 16S analysis, and includes *Defluviicoccus* Kalu MAXAC 148, but not the Bin 129 genome, and is consistent with phylogenetic analyses conducted using the more sparsely sampled whole genome datasets. As discussed above, we anticipate further delineation of the taxonomic structure of genus *Defluviicoccus*, as a consequence of the increasing numbers of both complete genome sequences, and full-length ribosomal SSU-rRNA sequences, that are becoming available (Dueholm et al., 2020; Arumugam et al., 2021; Singleton et al., 2021). The data presented here have confirmed much, but not all, of the earlier data generated for the then recognized clades by experimental methods (Burow et al., 2007, 2008a,b; Wong and Liu, 2007). Comparative analysis against 16S rRNA sequences from all members of the order *Rhodospirillales* does not support the view that *Defluviicoccus* should be moved into the family Geminicoccaceae, but is consistent with the notion that *Defluviicoccus* forms a distinct family within the *Rhodospirillales*.

Our interpretations are reliant on the quality of genome sequences included in this analysis. Of the genomes analyzed, only two are complete, closed (single chromosome) sequence, namely, those of *D. vanus*^T^ and *Defluviicoccus* SSA4, with the remainder being fractionated with contig numbers varying between 11 and 605 (Table 1). With the exception of the bin 129 genomes, all other genomes analyzed here are classified as high-quality draft genomes (as defined by the accepted MIMAG criteria). Notwithstanding that in the case of metagenome-assembled genomes (MAG), such fractionated genomes may harbor contaminant sequences (false positives) from non-cognate genomes and/or remain incomplete from the failure to incorporate all cognate sequences in the draft genome (false negatives), and both may occur in some of the genome sequence data described here.

Interestingly, the bin 129 genome derives from a marine, not wastewater source, and this might contribute to its substantive differences with the other eight, resulting from the imposition of quite different selection pressures. Alternatively, the distant position of sequence bin 129 in both the 16S rRNA and genome phylograms suggests that this genome arises from another group within the *Rhodospirillaceae* (Figure 4). Notably, the bin 129 genome lacks the genes considered to define the GAO phenotype, with none detected encoding the pathways for glycogen biosynthesis or degradation. At an estimated 86% completeness and <1% contamination, the bin 129 genome is close to being assessed as “high quality” and so the absence of genes for those pathways is probably real and does not result from false negatives associated with draft genome incompleteness.

Although these phylogenomic data support the presence of the extant metabolic features expected of populations possessing the GAO phenotype, one striking feature of their genomes is the functional diversity among them, especially in terms of their substrate preferences and possession of the *Pit* gene. This is not surprising given the similar clade diversity known to occur in both *Ca*. Accumulibacter (Flowers et al., 2013; Kolakovic et al., 2021) and *Ca*. Competibacter (McIlroy et al., 2014). Our analysis of glucan metabolism (glycogen and trehalose) shows that both classical (*glgC-glgA*) and non-classical pathways (*treS-pep2-glgE*) are present (Chandra et al., 2011); it remains open as to whether some members possess additional alternative pathways (i.e., the Kalu MAXAC 148 genome contains the *galU-otsA-otsB* trehalose biosynthesis system, not observed in the other genomes), as seen, for example, in members of the genus *Mycobacterium* (Koliwer-Brandl et al., 2016). A related area of exploration should involve possible interrelationships between intracellular and extracellular forms of glucan polymers, which may shed more light on their mechanisms of ecophysiological adaptation.

These functional variations are seen even among members of the same clade and serve to emphasize again how dangerous it is to make generalized predictions of their behavior in wastewater treatment plants. Consequently, any studies involving them should identify unequivocally which *Defluviicoccus* clade member is under examination, which is now possible from full-length 16S rRNA amplicon sequencing (Dueholm et al., 2020). The data here and those of Dueholm et al. (2020) should permit new, highly targeted FISH probes to be designed to cover the increasing diversity of *Defluviicoccus* now known to exist.

The analysis of the gene content of the *D. vanus*^T^ plasmid sequence clearly suggests that it is classifiable as a conjugative plasmid, based on the presence of gene clusters for its replication and propagation (Norman et al., 2009). The plasmid also contained at least one metal resistance operon (*czc*), which may convey tolerance to mercury. Recently Ma et al. (2019) have documented the presence of a *Defluviicoccus* population in mercury-contaminated soil, although its precise identity remains unknown.

Whether the sequence data can provide clues on how each of these organisms might be better grown in axenic condition remains to be seen: the advantage of having pure cultures of these and other strains is of paramount value. For example, there would be no need to rely on highly enriched populations in attempts to understand *in situ* physiology under different conditions, and the generation of genome sequence data would be less equivocal than in the case of metagenome-assembled genomes. With pure cultures, it would be easier to elucidate the factors affecting regulation of enzyme synthesis and activity of key enzymes, using transcriptome and proteomic approaches, and interpretation of complementary functional assays, such as FISH/MAR (Carr et al., 2006), nanoSIMS (Musat et al., 2012), or Raman-FISH spectroscopy (Huang et al., 2007; Fernando et al., 2019). For example, the difficulty experienced growing *D. vanus*^T^ on GS medium, with glucose, chosen somewhat arbitrarily as its carbon source (Maszenan et al., 2005), may be overcome by replacing it with either acetate and or propionate under alternating anaerobic/aerobic conditions and fine-tuning medium composition based on its gene complement and known phenotype.

## Data Availability Statement

The *D. vanus* chromosomal and plasmid genomes are available via GenBank accessions CP053923.1 and CP053924.1, respectively. The corresponding raw sequence data is being released via NCBI BioProject accession PRJNA635277.

## Author Contributions

RS proposed the study. IB, AM, and RW designed the experiments. IB and NS performed experimental work. IB performed long read sequencing. IB, AM, RS, and RW designed the analyses. IB, MH, KA, and RW performed data analysis. All authors were involved in data interpretation. RS and RW primarily wrote the manuscript, with inputs from other authors in specific areas.

## Conflict of Interest

The authors declare that the research was conducted in the absence of any commercial or financial relationships that could be construed as a potential conflict of interest.

## Publisher’s Note

All claims expressed in this article are solely those of the authors and do not necessarily represent those of their affiliated organizations, or those of the publisher, the editors and the reviewers. Any product that may be evaluated in this article, or claim that may be made by its manufacturer, is not guaranteed or endorsed by the publisher.

## Footnotes

1
https://github.com/rbhwilliams/RKXM

2
imedea.uib-csic.es/mmg/ltp/

3
https://www.arb-silva.de/aligner/

## Supplementary Material

Supplementary Figure 1 GC-coverage plot of the short read assembly from *Defluviicoccus vanus^T^*. The rectangular region delineated with dashed lines contains the contigs considered to arise from the chromosomal genome, which is denoted as bin 0.2 in the analysis presented in Supplementary Figure 2.

Supplementary Figure 2 Summary of the concordance-statistic analysis between the *D. vanus* genome recovered from long read sequence data (denoted as LR-chr 1) and the *D. vanus* genome recovered short read data (denoted as bin 0.2; see Supplementary Figure 1). Both sets of sequence were obtained from the same DNA aliquot. The concordance statistic (κ, *kappa*) was computed using the srac2lrac from BLASTN alignments (see Section “Methods: Short Read Sequencing and Data Analyses and Arumugam et al. (2021) for Further Details]. **(A)** Distribution of κ-statistics from comparison of LR-chr 1 against genome bins recovered from the corresponding short read assembly; the highest scoring genome bin (bin 0.2; see Supplementary Figure 1) is indicated, indicating the high degree of recapitulation between each draft genome. Other statistics shown arise from alternative bins shown in Supplementary Figure 1. **(B)** Coverage-GC plot for the short read assembly, with bin 2 highlighted (closed black circles and dark gray convex hull; other bins are highlighted by light gray convex hulls). **(C)** Short read (SR: black crosses) and long read (LR: gray crosses) coverage profiles across LR-chr 1, showing expected uniform coverage across LR-chr 1. **(D–F)** BLASTN statistics for alignments of short read contigs from bin 0.2 against LR-chr 1. Horizontal segments show alignment position on the long read sequence (horizontal axis) and the height of the segment is value of corresponding statistic (vertical–axis) namely percent identity (PID) in **(D)**, the ratio of alignment length to query length (al2ql) in **(E)** and log*_10_* bitscore in **(F)**. Note the presence of full length, high quality alignments of contig sequence from bin 0.2 to LR-chr 1. **(G)** GC content as a function of position on LR-chr 1 (gray closed circles, computed in adjacent windows of length 46,700 bp) and for aligned short read contigs (black closed circles), showing highly correlated patterning of GC content between the two sets of sequences. **(H–K)** Distribution of four component statistics of κ (refer Arumugam et al., 2021), with the position of the top scoring short read bin (bin 0.2) highlighted. **(H)** Proportion of short read contigs in bin aligned to LR-chr 1 (*p*_*srac*_). **(I)** Mean percent identity (*pid*) and **(J)** mean ratio of alignment length to query length (*al2ql*) and **(K)** proportion of the long read contig (LR-chr 1) that is covered by an alignment from bin 0.2 (*p*_*aln*_). Approximately 10% of the LR-chr 1 sequence is not covered by aligned short read contigs, most likely due to incompleteness of the latter genome bin.

Supplementary Figure 3 Whole genome sequence phylogram from genomes from genus *Defluviicoccus* (*n* = 9*;* Table 1) *and genomes from members of order Rhodospirillales* (*n* = 85) available in the GTDB database. Whole genome distance statistics were computed within GTDB-Tk. Following Onetto et al. (2019), genomes from members of the genus *Gemmatimonas* (*n* = 5) were assigned as the outgroup taxa. See Section “Materials and Methods: Comparative Analyses of *Defluviicoccus* genomes” for details.

Supplementary Figure 4 Network representation of the occurrence of genes in the KEGG among the genomes from genus *Defluviicoccus*. Genes are shown as red nodes and genomes are shown as blue nodes. Edges are weighted by the copy number of the gene in the cognate genome, in this case ranging from one copy to three copies. Full node names as follows; *307*: *Ca.* Defluviicoccus Fred MAXAC 307; *378*, Ca. Defluviicoccus Fred MAXAC 378; *HK*, Defluviicoccus GAO-HK; *148*, *Ca*. Defluviicoccus Kalu MAXAC 148; *SSA4*, *Ca*. Defluviicoccus. sp. SSA4; *129*, Defluviicoccus bin 129; *TF071*, Ca. Defluviicoccus tetraformis TF071; *Vanus*, *Defluviicoccus vanus^T^*; *narG*, nitrate reductase, alpha subunit; *narI*, respiratory nitrate reductase 1 gamma chain; *narY*, respiratory nitrate reductase 2 beta chain; *nirK*, copper-containing nitrite reductase; *nirS*, nitrite reductase; *norB*, nitric oxide reductase, subunit B; *norC*, nitric oxide reductase, subunit B.

Supplementary Data File 1 Combined functional annotations for all protein-coding genes in the nine *Defluviicoccus* genomes listed in Table 1. Source data from Prokka and BlastKOALA as specified in the column names.

Supplementary Data File 2 Combined functional annotations for all protein-encoding genes in the plasmid sequence recovered from *Defluviicoccus vanus^T^*. Source data from Prokka and BlastKOALA as specified in the column names.

Supplementary Data File 3 BLASTN analysis of the plasmid sequence covered from *D. vanus* against the NCBI nt database.

Supplementary Data File 4 Sequence alignment results relating to the identification of *pep*2-*treS fused protein sequence*; see legend for Figure 6. Protein sequence annotated to *pep2* were sourced from the .faa files generated by Prokka in each of the 9 genomes listed in Table 1. In all cases except *D. vanus*, where a single adjacent pair of *treS* (*D. vanus* gene: CDGBEKEE_01737; length 1,764 bp) and *pep2*/*mak* (CDGBEKEE_01738; length 1,491 bp) were detected, in the remaining genomes *pep2*/*mak* was absent and *treS* was approximately 3,300 bp in length (the sole exception being the Kalu 148 genome, in which a second copy of *treS* was present, being 1,617 bp in length, but without a flanking *pep2*/*mak* gene). Herein these are referred to “long” *treS* sequences. Given the consistency of these observations with the common occurrence of “fused” *treS-pep2* sequence documented by Chandra et al. (2011), we tested the hypothesis that the long versions of *treS* contained the *pep2/mak* using BLASTP with the *D. vanus pep2/mak* protein sequence as a query and the complete set of 10 *treS* sequences as the subjects. The resulting alignment are provided in the this file; clearing showing that in the case of the long *treS* sequences, the *D. vanus pep2/mak* is highly aligned with distal 40% of the *treS* sequence, consistent with analysis of Chandra et al. (2011).
