# Supplementary material for: Comparative Genomics of Members of the Genus Defluviicoccus With Insights Into Their Ecophysiological Importance
Source: Front Microbiol. 2022 Apr 12;13:834906. doi: 10.3389/fmicb.2022.834906 (PMC9041414; doi:10.3389/fmicb.2022.834906)
Supplement: Supplementary Figure 1 — GC-coverage plot of the short read assembly from Defluviicoccus vanusT. The rectangular region delineated with dashed lines contains the contigs considered to arise from the chromosomal genome, which is denoted as bin 0.2 in the analysis presented in Supplementary Figure 2. [file Presentation_1.pdf]

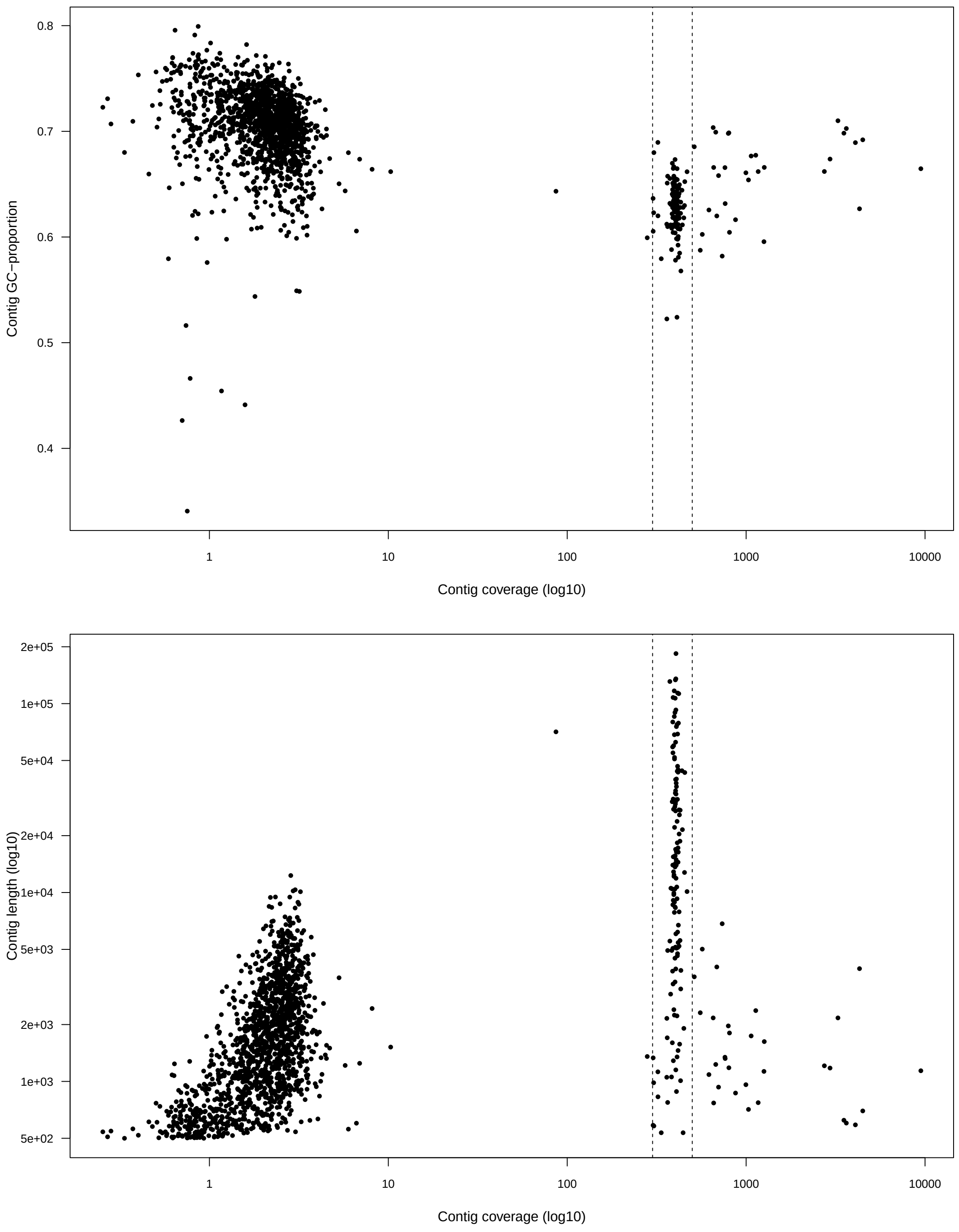

Supplementary Figure 1: Bessarab *et al.* 2021

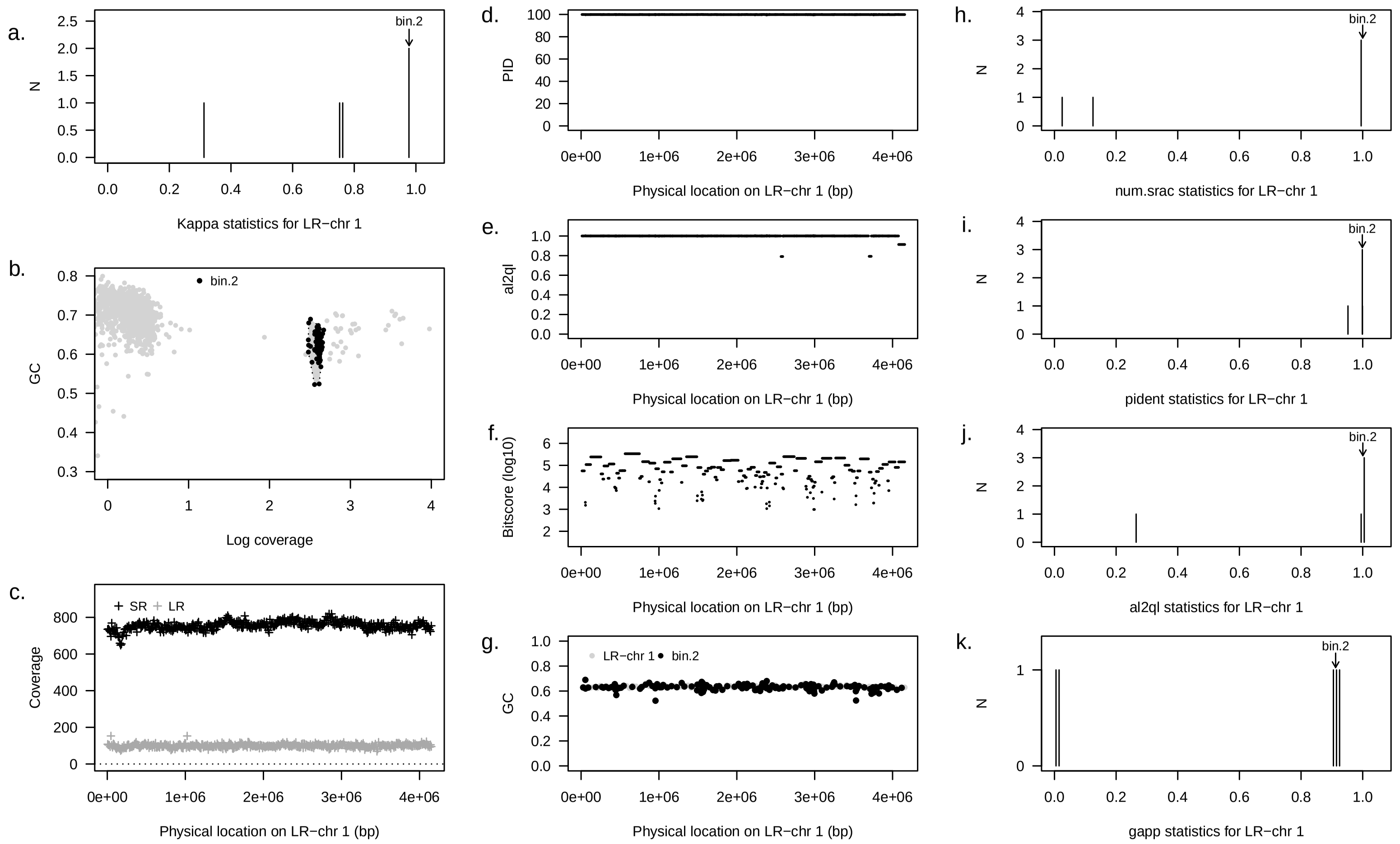

Supplementary Figure 2: Bessarab *et al.* 2021

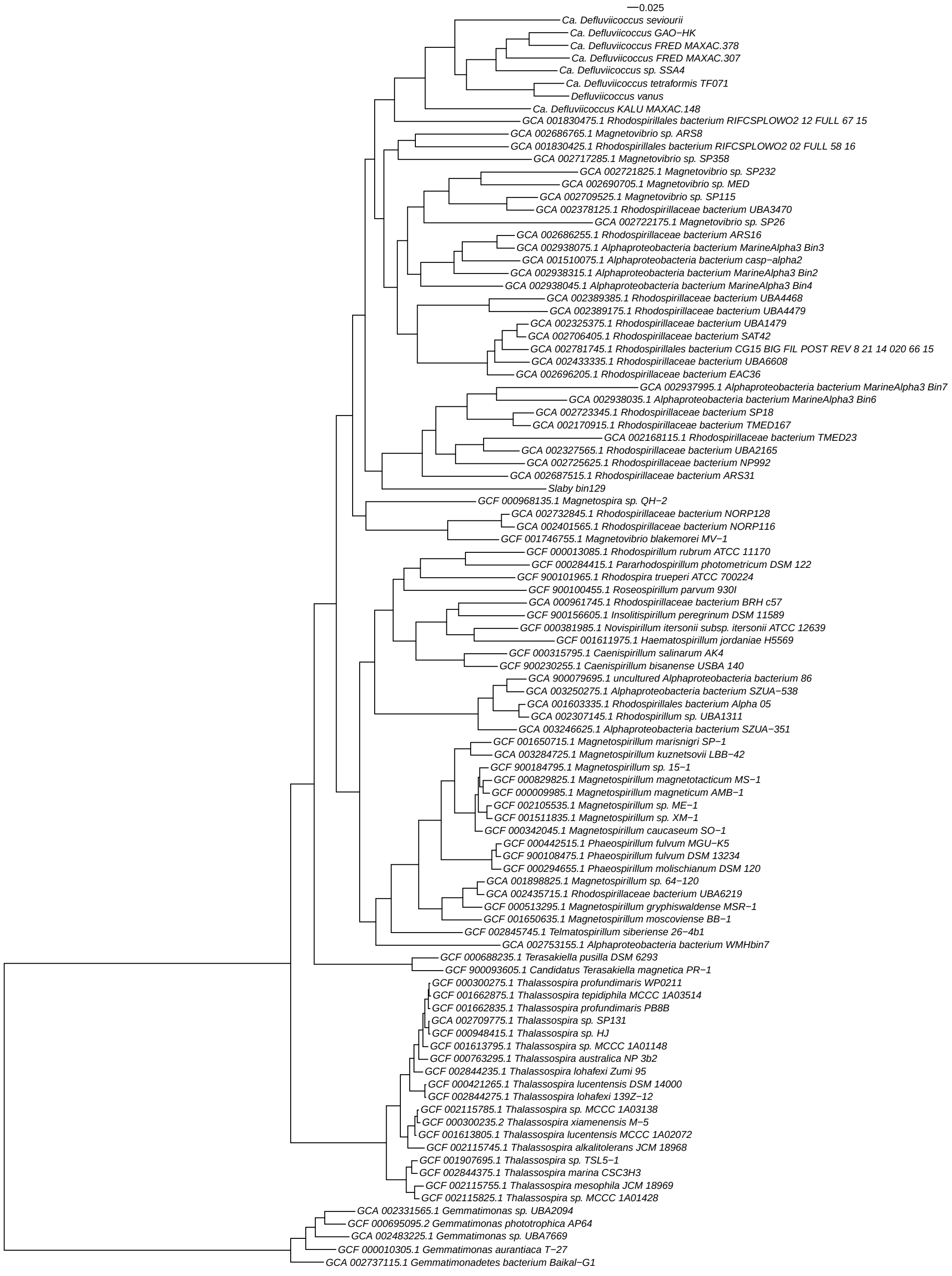

Supplementary Figure 3: Bessarab *et al.* 2021

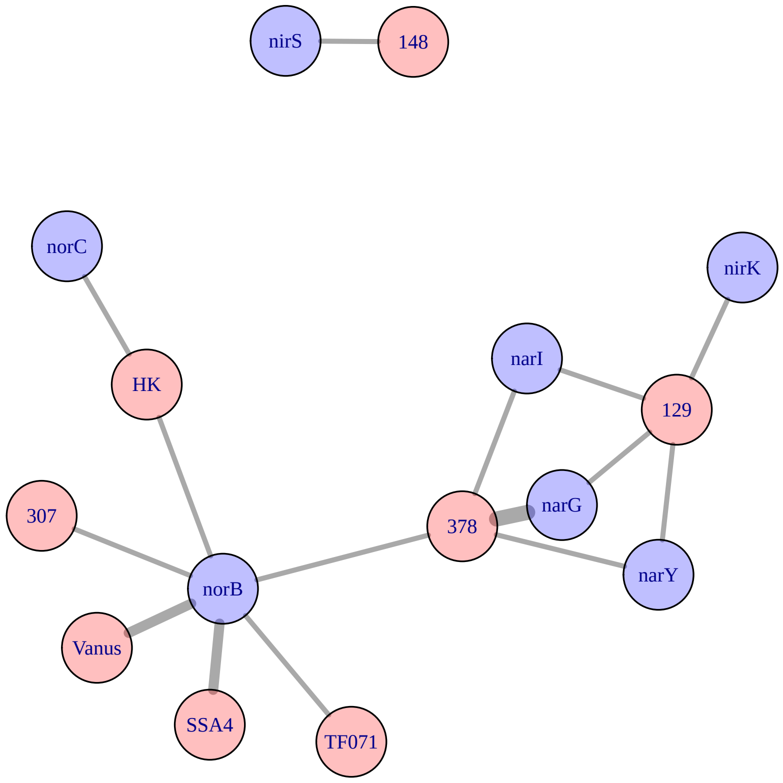

Supplementary Figure 4: Bessarab *et al.* 2021
