# Supplementary material for: Comparative Genomics of Members of the Genus Defluviicoccus With Insights Into Their Ecophysiological Importance
Source: Front Microbiol. 2022 Apr 12;13:834906. doi: 10.3389/fmicb.2022.834906 (PMC9041414; doi:10.3389/fmicb.2022.834906)
Supplement: Supplementary Data File 4 — Sequence alignment results relating to the identification of pep2-treS fused protein sequence; see legend for Figure 6. Protein sequence annotated to pep2 were sourced from the .faa files generated by Prokka in each of the 9 genomes listed in Table 1. In all cases except D. vanus, where a single adjacent pair of treS (D. vanus gene: CDGBEKEE_01737; length 1,764 bp) and pep2/mak (CDGBEKEE_01738; length 1,491 bp) were detected, in the remaining genomes pep2/mak was absent and treS was approximately 3,300 bp in length (the sole exception being the Kalu 148 genome, in which a second copy of treS was present, being 1,617 bp in length, but without a flanking pep2/mak gene). Herein these are referred to “long” treS sequences. Given the consistency of these observations with the common occurrence of “fused” treS-pep2 sequence documented by Chandra et al. (2011), we tested the hypothesis that the long versions of treS contained the pep2/mak using BLASTP with the D. vanus pep2/mak protein sequence as a query and the complete set of 10 treS sequences as the subjects. The resulting alignment are provided in the this file; clearing showing that in the case of the long treS sequences, the D. vanus pep2/mak is highly aligned with distal 40% of the treS sequence, consistent with analysis of Chandra et al. (2011). [file Data_Sheet_4.pdf]

```
BLASTP 2.4.0+


Reference: Stephen F. Altschul, Thomas L. Madden, Alejandro A.
Schaffer, Jinghui Zhang, Zheng Zhang, Webb Miller, and David J.
Lipman (1997), "Gapped BLAST and PSI-BLAST: a new generation of
protein database search programs", Nucleic Acids Res. 25:3389-3402.


Reference for composition-based statistics: Alejandro A. Schaffer,
L. Aravind, Thomas L. Madden, Sergei Shavirin, John L. Spouge, Yuri
I. Wolf, Eugene V. Koonin, and Stephen F. Altschul (2001),
"Improving the accuracy of PSI-BLAST protein database searches with
composition-based statistics and other refinements", Nucleic Acids
Res. 29:2994-3005.



Database: treS_defluviivoccus.faa
           10 sequences; 9,915 total letters



Query= CDGBEKEE_01738

Length=496
                                                                      Score     E
Sequences producing significant alignments:                          (Bits)  Value

  HKIMANAB_01080                                                      540     0.0
  AMCANFMH_01459                                                      477     3e-161
  CLOEAMAL_01136                                                      427     7e-142
  IOOFFHIP_03803                                                      424     5e-141
  AILEONCL_00133                                                      418     1e-138
  FLNIPHHM_03499                                                      409     4e-135
  MKNNJHOE_00428                                                      399     2e-131
  AMCANFMH_01079                                                      328     8e-105
```

```
> HKIMANAB_01080
Length=1101

 Score = 540 bits (1391),  Expect = 0.0, Method: Compositional matrix adjust.
 Identities = 284/485 (59%), Positives = 350/485 (72%), Gaps = 1/485 (0%)

Query  4     IADHVDFTAETDGCVLMFLNVAREETSAQRYALPLALAWESASDDPLARLQAQVVARVRR  63
               AD V   + +  C + F++V+RE    QRY+LPLA+AWE+  D+PL+RLQ  V+ARVRR
Sbjct  614   FADRVHLVSGSGDCAIAFIDVSREGAPFQRYSLPLAIAWETNDDEPLSRLQPNVLARVRR  673

Query  64    GSHLGVLYDATASDELPRLLIDAIRGGAELPTRGGARLVCRSTGALTAYSDVNTSNCRRL  123
             G H+GVLYDAT  DELP LL +A+  G ++ +   + L CR T A  A+ DV+  NCRRL
Sbjct  674   GPHVGVLYDATVGDELPLLLFEAVGAGQDIRSAANSCLACRPTAAFEAFRDVSLKNCRRL  733

Query  124   GVEQSNTSMLIDDQMIIKLYRRLQSGIHPEVEIGHFLTSVAGYTSAPALLGSVELLDDDG  183
             GVEQSNTS+LIDD++IIK+YRRLQ+G HPE+EIG FLT VA Y +AP LLGSVELL  DG
Sbjct  734   GVEQSNTSLLIDDRLIIKIYRRLQAGTHPEIEIGRFLTDVAHYANAPRLLGSVELLGGDG  793

Query  184   SVTAIAVLQEFVRNQGDGWEMTLDHLERVLNQLDLTLLSAED-YDDEAAHESYWASVATL  242
             SVTA+A++QEFVRNQGDGW MTLDHL+RVLN++ +   +  D +DDE AHE YW    TL
Sbjct  794   SVTAVALVQEFVRNQGDGWAMTLDHLDRVLNEVGMMPPTESDVFDDERAHEGYWLLTETL  853

Query  243   ARRVGELHLAFALTVDDPAFAPEPTSEDDIATWNRQIAELATKAHRILESAVHADALGEE  302
             A R+G+LH A A+  DD AFAPEP    DI  W  QIA LA KA   L  AV    LG E
Sbjct  854   AGRIGDLHRALAMDTDDRAFAPEPAGPSDIQAWTGQIASLAGKARDTLGRAVAEGKLGGE  913

Query  303   ARSLGTQLLASWPAIEALATIPATTLTGIMKTRIHGDLHLGQVVVAGTNFFILDFEGEPL  362
             AR+L  + L  W  IE L  +PA  L G +KTRIHGDLHLGQVVVAGT+F++LDFEGEPL
Sbjct  914   ARALAERTLDDWGMIERLGVVPADALAGTVKTRIHGDLHLGQVVVAGTDFYVLDFEGEPL  973

Query  363   RTLEARRQXSFPLRDVAGLLRSFDYAGSAALIRRGSIANAEKGVASERDAITKWRRETSA  422
               LE RR  S PLRDVAG++RSFDYAG+ A  +R S A + +G A ER  I +WR  T+
Sbjct  974   HGLERRRAKSSPLRDVAGMVRSFDYAGNTAANKRKSPAASGEGAALERATIARWRTTTTR  1033

Query  423   RFMSTYRETVADCAALPQAEEAFVAALDAFVIEKALYEICYEAANRPDWLSIPLAGVHRL  482
```

```
             RF++ YR   A C ++PQ + AF AA+DAFV+EKALYE+CYEAANRPDWL IPLAG+ RL
Sbjct  1034  RFLAAYRGASAGCLSVPQDDAAFAAAVDAFVLEKALYEVCYEAANRPDWLGIPLAGIARL  1093

Query  483   LETVR  487
             L+  +
Sbjct  1094  LDNAK  1098


> AMCANFMH_01459
Length=1096

 Score = 477 bits (1228),  Expect = 3e-161, Method: Compositional matrix adjust.
 Identities = 247/486 (51%), Positives = 327/486 (67%), Gaps = 11/486 (2%)

Query  2     VGIADHVDFTAETDG-CVLMFLNVAREETSAQRYALPLALAWESASDDPLARLQAQVVAR  60
             VGIA+ V F A   G C + F++V  E  + Q Y+LPL +AWESA+DDPL RLQ  V+AR
Sbjct  608   VGIANWVGFGAGAAGECGIAFIDVDSEGQAPQSYSLPLGIAWESATDDPLQRLQPMVLAR  667

Query  61    VRRGSHLGVLYDATASDELPRLLIDAIRGGAELPTRGGARLVCRSTGALTAYSDVNTSNC  120
             VRR + +GVL+DA AS EL + + DA+   AE+   GG RLVC+ST A   +SD+    C
Sbjct  668   VRRAASVGVLHDAAASPELAKTIFDAVARNAEIDGPGG-RLVCKSTAAFAEFSDIAPEQC  726

Query  121   RRLGVEQSNTSMLIDDQMIIKLYRRLQSGIHPEVEIGHFLTSVAGYTSAPALLGSVELLD  180
              RLGVEQSNTS L+D+++++K YRR+Q GIHPEVEIG FLT VA Y + P LLGS+EL+
Sbjct  727   ARLGVEQSNTSTLLDNRVVLKFYRRVQVGIHPEVEIGRFLTDVALYANVPRLLGSIELIS  786

Query  181   DDGSVTAIAVLQEFVRNQGDGWEMTLDHLERVLNQLDLTLLSAEDYDDEAAHESYWASVA  240
              DG  TA+A+LQEFVRNQGD W  TL+HL+R LN ++L L  +E   D+  H  YW  V
Sbjct  787   ADGEATAVAMLQEFVRNQGDAWMFTLEHLDRTLNAVELLLPGSEHVGDD-PHAGYWVLVE  845

Query  241   TLARRVGELHLAFA-LTVDDPAFAPEPTSEDDIATWNRQIAELATKAHRILESA--VHAD  297
             T+ARR+GELH+A A     DPAFAPEP S  +   W  ++ E+A +A   + +A     D
Sbjct  846   TMARRIGELHIALANAGSGDPAFAPEPMSIAERDRWQNELLEIAMRARTAVMTARTRQKD  905

Query  298   ALGEEARSLGTQLLASWPAIEALATIPATTLTGIMKTRIHGDLHLGQVVVAGTNFFILDF  357
                EE   L T +L+ W  ++ + T+P + +  +  TR+HGDLHLGQVVVAG++ ++LDF
Sbjct  906   PRIEE---LATHILSYWEVLQKICTLPGSAIVSVASTRVHGDLHLGQVVVAGSDCYLLDF  962
```

```
Query  358   EGEPLRTLEARRQXSFPLRDVAGLLRSFDYAGSAALIRRGSIANAEKGVASERDAITKWR  417
             EGEPLR ++ RR  + PLRDVAG++RSF+YAG  AL RRGS   A +  A  R AI  W
Sbjct  963   EGEPLRPIQTRRAKTSPLRDVAGMIRSFEYAGETALARRGSGPVAPR--ADGRKAIDTWL  1020

Query  418   RETSARFMSTYRETVADCAALPQAEEAFVAALDAFVIEKALYEICYEAANRPDWLSIPLA  477
              +T  RF++ YRET  +C ++P   + F   LD F +EK LYE+CYEAANRPDWL IPL+
Sbjct  1021  EQTKLRFIAAYRETTLNCMSVPPVLDDFNMLLDVFTLEKTLYEVCYEAANRPDWLGIPLS  1080

Query  478   GVHRLL  483
              + + L
Sbjct  1081  ALAQRL  1086


> CLOEAMAL_01136
Length=1103

 Score = 427 bits (1097),  Expect = 7e-142, Method: Compositional matrix adjust.
 Identities = 236/486 (49%), Positives = 302/486 (62%), Gaps = 5/486 (1%)

Query  2     VGIADHVDFTAETDGCVLMFLNVAREETSAQRYALPLALAWESASDDPLARLQAQVVARV  61
             V IAD    + + +  +L+ L+   E   AQRY LPLA+AWESA DDP ARL +  +AR
Sbjct  610   VAIADTAVLSHDGEAFLLLTLDAFIESGEAQRYLLPLAIAWESAIDDPFARLHSYTLARA  669

Query  62    RRGSHLGVLYDATASDELPRLLIDAIRGGAELPTRGGARLVCRSTGALTAYSDVNTSNCR  121
             R G  +G ++DA A   L R ++  +R G  +PTR   RL+ R T A      +   +
Sbjct  670   RTGGRIGAIHDAVAGPGLARAVVAGVRDGLTVPTRDDGRLIFRRTKAFPEPLPLAEISVE  729

Query  122   RLGVEQSNTSMLIDDQMIIKLYRRLQSGIHPEVEIGHFLTSVAGYTSAPALLGSVELLDD  181
             R+G EQSNTS+ + +++I+KL RRL  G+HPEVE+G FLT VAGYT+ P LLGSVE +D
Sbjct  730   RMGGEQSNTSLKLGEEIILKLLRRLAPGVHPEVEVGRFLTEVAGYTNVPPLLGSVEHIDA  789

Query  182   DGSVTAIAVLQEFVRNQGDGWEMTLDHLERVLNQLDLTLLSAEDYDDEAAHESYWASVAT  241
             +G  TA+ VLQ FVRNQGDGWE TLDHL+RVL QL+  L S  D D    +  +     T
Sbjct  790   EGVPTALVVLQGFVRNQGDGWEFTLDHLDRVLGQLEF-LPSGVDADPAELYGGFLPLAET  848

Query  242   LARRVGELHLAFALTVDDPAFAPEPTSEDDIATWNRQIAELATKAHRILESAVHADALGE  301
```

```
             L RR+ ELH AFAL  DD AF  EP    DI   W  + + A  AH  L  AV+ D+LGE
Sbjct  849   LGRRIAELHRAFALPTDDAAFRSEPAGPADIEAWVAAVRQQAASAHEALTRAVN-DSLGE  907

Query  302   EARSLGTQLLASWPAIEALATIPATT-LTGIMKTRIHGDLHLGQVVVAGTNFFILDFEGE  360
               R+    LL  W  IE+ A + +   + G++KTR+HGDLHLGQVVV   +F ILDFEGE
Sbjct  908   LVRAEVQALLDRWGEIESAARVTSDRGIEGLVKTRLHGDLHLGQVVVVRDDFHILDFEGE  967

Query  361   PLRTLEARRQXSFPLRDVAGLLRSFDYAGSAALIRRGSIANAEKGVASERDAITKWRRET  420
             PLR L  RR  S PLRDVAG+LRSFDYA S AL RR   +     G    R A+  WR +
Sbjct  968   PLRPLSERRVKSSPLRDVAGMLRSFDYAASTALHRRPEVRPG--GGEVLRRALLDWRHQV  1025

Query  421   SARFMSTYRETVADCAALPQAEEAFVAALDAFVIEKALYEICYEAANRPDWLSIPLAGVH  480
               +F+  Y   +A C ++P AE  F + LD F++EK LYEI YEAANRPDWL IP+ G+
Sbjct  1026  IGQFLEGYWTAIAGCPSVPAAEATFRSMLDLFLLEKVLYEIRYEAANRPDWLIIPVGGLR  1085

Query  481   RLLETV  486
              LL+ +
Sbjct  1086  ALLDRI  1091


 Score = 21.6 bits (44),  Expect = 1.2, Method: Compositional matrix adjust.
 Identities = 14/38 (37%), Positives = 20/38 (53%), Gaps = 1/38 (3%)

Query  417  RRETSARFMSTYRETVADCAALPQAEEAF-VAALDAFV  453
            +R  +A+     R  +AD A L    EAF +  LDAF+
Sbjct  597  QRWFAAKGQRIERVAIADTAVLSHDGEAFLLLTLDAFI  634


> IOOFFHIP_03803
Length=1102

 Score = 424 bits (1091),  Expect = 5e-141, Method: Compositional matrix adjust.
 Identities = 229/482 (48%), Positives = 295/482 (61%), Gaps = 4/482 (1%)

Query  2     VGIADHVDFTAETDGCVLMFLNVAREETSAQRYALPLALAWESASDDPLARLQAQVVARV  61
             + +AD      + D  +++ L+V+ +    QRY LPLA+AWESASDDP ARLQ+  +AR
Sbjct  607   IAVADTAVLPGDGDDFLMLTLDVSLDGGEVQRYLLPLAIAWESASDDPFARLQSYTLARA  666
```

```
Query  62    RRGSHLGVLYDATASDELPRLLIDAIRGGAELPTRGGARLVCRSTGALTAYSDVNTSNCR  121
             R G  +G ++DA  + E  R +  A+R G  LPTR G +LV + T A      +
Sbjct  667   RTGGRIGAIHDAALAPEFARAVAVAVRDGLALPTRSGGQLVFQPTSAFPRDLPLAGIAVD  726

Query  122   RLGVEQSNTSMLIDDQMIIKLYRRLQSGIHPEVEIGHFLTSVAGYTSAPALLGSVELLDD  181
             RLG EQSNTS+L+   +I+K  RRL SG+HPEVEIG FLT VA + + P L GSVE+ D
Sbjct  727   RLGREQSNTSLLLGADIILKALRRLASGVHPEVEIGRFLTEVARFPNTPPLFGSVEMRDG  786

Query  182   DGSVTAIAVLQEFVRNQGDGWEMTLDHLERVLNQLDLTLLSAEDYDDEAAHESYWASVAT  241
             DG  T++  LQ FVRNQGDGWE TLDHL+R+L QL+  L S  D D    +E Y     T
Sbjct  787   DGVPTSLLTLQGFVRNQGDGWEFTLDHLDRLLGQLEF-LPSGVDADLGELYEVYLTMAET  845

Query  242   LARRVGELHLAFALTVDDPAFAPEPTSEDDIATWNRQIAELATKAHRILESAVHADALGE  301
             L RRV ELH AFAL VDDPAF P+P +  D+  W   +   A  AH  L  A+ +  LGE
Sbjct  846   LGRRVAELHRAFALPVDDPAFHPDPANAADVTAWGEAVLRQAQSAHAALTRALES-GLGE  904

Query  302   EARSLGTQLLASWPAIEALATIPATTLTGIMKTRIHGDLHLGQVVVAGTNFFILDFEGEP  361
             +       LL  W +++A          G +KTR+HGDLHLGQVVV   +F I+DFEGEP
Sbjct  905   DVAVAVRSLLDRWQSVQAAVAAVHGHDFGFVKTRVHGDLHLGQVVVVRDDFDIIDFEGEP  964

Query  362   LRTLEARRQXSFPLRDVAGLLRSFDYAGSAALIRRGSIANAEKGVASERDAITKWRRETS  421
             LR+LE RR    PL+DVAG+LRSF YA S AL RR  +     G    R  +  WRR+
Sbjct  965   LRSLEERRCKGSPLKDVAGMLRSFHYAASTALHRRPEVRPG--GGEMLRQTLEGWRRKVV  1022

Query  422   ARFMSTYRETVADCAALPQAEEAFVAALDAFVIEKALYEICYEAANRPDWLSIPLAGVHR  481
              RF+  YR  + DC ++P  +  F A L+ F++EK LYEICYEAANRPDWL+IP+ G
Sbjct  1023  GRFLQGYRTAIGDCPSVPADDAEFHALLELFMLEKVLYEICYEAANRPDWLAIPVNGALA  1082

Query  482   LL  483
             +L
Sbjct  1083  ML  1084


> AILEONCL_00133
Length=1101
```

```
 Score = 418 bits (1075),  Expect = 1e-138, Method: Compositional matrix adjust.
 Identities = 227/469 (48%), Positives = 297/469 (63%), Gaps = 5/469 (1%)

Query  16    GCVLMFLNVAREETSAQRYALPLALAWESASDDPLARLQAQVVARVRRGSHLGVLYDATA  75
             G ++    VA     A RY +PLA AWE+A DDPL RLQ+  +A+VR G+ +GVLYDA A
Sbjct  630   GYLIATFQVATRGGGAYRYHMPLAAAWETAVDDPLQRLQSFTLAKVRTGNRVGVLYDALA  689

Query  76    SDELPRLLIDAIRGGAELPTRGGARLVCRSTGALTAYSDVNTSNCRRLGVEQSNTSMLID  135
                    ++++IRGG E+ T  G  +   +T A  A          R+G EQSNTS+ I
Sbjct  690   DAPFTAAVLESIRGGREMGTANGGTIRFAATNAFAAVEIPADVAIDRMGGEQSNTSLKIG  749

Query  136   DQMIIKLYRRLQSGIHPEVEIGHFLTSVAGYTSAPALLGSVELLDDDGSVTAIAVLQEFV  195
             D  I+K YRRL++G HPE+E+G FLT VAGY + P LLGSVEL D DG+ TA+AVL  FV
Sbjct  750   DVAILKGYRRLEAGSHPELEMGRFLTDVAGYANTPPLLGSVELFDADGTPTALAVLHGFV  809

Query  196   RNQGDGWEMTLDHLERVLNQLDLTLLSAEDYDDEAAHESYWASVATLARRVGELHLAFAL  255
             RNQGDGW  T+D+L+R ++++++T +  E    E  H  Y A  ATL  R+ ELH A AL
Sbjct  810   RNQGDGWSFTVDYLDRHISEIEVTPVGDEPA-PEQPHALYHALAATLGTRIAELHAALAL  868

Query  256   TVDDPAFAPEPTSEDDIATWNRQIAELATKAHRILESAVHADALGEEARSLGTQLLASWP  315
              VDDPAF PEP S++D+A W  Q+   A  AH+ L  A+  DAL ++ R +   +L  WP
Sbjct  869   DVDDPAFRPEPASDEDLAQWGEQVRRQAAAAHQALTRAMAQDALTDDVREMVGSVLERWP  928

Query  316   AIEALATIP-ATTLTGIMKTRIHGDLHLGQVVVAGTNFFILDFEGEPLRTLEARRQXSFP  374
             AI+ +      +   G+MK+RIHGDLHLGQVVV   +F++LDFEGEP + +E RR    P
Sbjct  929   AIDGMVEQSFGSAGEGLMKSRIHGDLHLGQVVVVREDFYVLDFEGEPAKAMEHRRTKQSP  988

Query  375   LRDVAGLLRSFDYAGSAALIRRGSIANAEKGVASERDAITKWRRETSARFMSTYRETVAD  434
             LRDVAG++RSFDYAG AA   R       + VA    A+ +W  + +  F+  YR T A
Sbjct  989   LRDVAGMVRSFDYAGWAARAERLPTQTRVEDVAR---AVEEWTLQATECFLGAYRTTAAG  1045

Query  435   CAALPQAEEAFVAALDAFVIEKALYEICYEAANRPDWLSIPLAGVHRLL  483
             CA +P  + AF   LD F +EKALYEICYEA NRPDWL IP+ GV R+L
Sbjct  1046  CATVPGDDAAFQRLLDVFTLEKALYEICYEADNRPDWLRIPVQGVARIL  1094


> FLNIPHHM_03499
```

```
Length=1096

 Score = 409 bits (1051),  Expect = 4e-135, Method: Compositional matrix adjust.
 Identities = 232/489 (47%), Positives = 310/489 (63%), Gaps = 12/489 (2%)

Query  2     VGIADHVDFTAETDGCVLMFLNVARE----ETSAQRYALPLALAWESASDDPLARLQAQV  57
             + +AD V++ + ++  ++  ++         E   QRY LPLA+AWE+  DDP  RLQA
Sbjct  608   IDLADAVEYASGSEDFLIALIDAVMPTPGGEERRQRYLLPLAIAWETTQDDPFGRLQAFT  667

Query  58    VARVRRGSHLGVLYDATASDELPRLLIDAIRGGAELPTRGGARLVCRSTGALTAYSDVNT  117
             +AR R G  +G ++DA AS +  R ++   R   ELPT  G RL+ R T A   + D++
Sbjct  668   LARARIGGRIGAIHDAMASPQFARAVLIGARDQKELPTSRGGRLMFRRTKAFPLFDDIDA  727

Query  118   SNCRRLGVEQSNTSMLIDDQMIIKLYRRLQSGIHPEVEIGHFLTSVAGYTSAPALLGSVE  177
                 RLG EQSNTS+LI + +I+K +RR++ GIHPEVEIG FLT V+G+ +APALLGSVE
Sbjct  728   LAAGRLGREQSNTSVLIGEDVILKAFRRIEPGIHPEVEIGRFLTDVSGFENAPALLGSVE  787

Query  178   LLDDDGSVTAIAVLQEFVRNQGDGWEMTLDHLERVLNQLDLTLLSAEDYDDEAAHESYWA  237
             L+D DG  TAIAVLQ+FVRNQGDGW  TLD+L+R++ QL+  + + E      AH  Y A
Sbjct  788   LIDADGVPTAIAVLQKFVRNQGDGWGFTLDYLDRLVGQLEF-MPADEATQQVDAHVVYAA  846

Query  238   SVATLARRVGELHLAFALTVDDPAFAPEPTSEDDIATWNRQIAELATKAHRILESAVHAD  297
                TL RR+ E+H AFAL VDDPAF PEPTS  D+  W   + + A +A   LE A  A
Sbjct  847   LAETLGRRIAEMHKAFALPVDDPAFQPEPTSNADVIAWTDGVLDQARRAREDLEHA-RAS  905

Query  298   ALGEEARSLGTQLLASWPAIE-ALATIPATTLTGIMKTRIHGDLHLGQVVVAGTNFFILD  356
              L E         LL+ +  I+ A+A I   T   ++KTR+HGDLHLGQVVV   +F+ILD
Sbjct  906   GLNETLHQDVEDLLSRFADIDTAIARIRNQTYN-VVKTRVHGDLHLGQVVVVREDFYILD  964

Query  357   FEGEPLRTLEARRQXSFPLRDVAGLLRSFDYAGSAALIRRGSIANAEKGVASE-RDAITK  415
             FEGEPLR +  RR    PL+DVAG+LRSFDYA  +A+ RR  +     GVA   R  +
Sbjct  965   FEGEPLRPMAERRAKQSPLKDVAGMLRSFDYAAYSAVHRRPEV---RPGVAEVLRTEMAA  1021

Query  416   WRRETSARFMSTYRETVADCAALPQAEEAFVAALDAFVIEKALYEICYEAANRPDWLSIP  475
             W      +F++ YR  V  C ++P  + AF   L+ F++EKALYE+CYEAANRPDWL+IP
Sbjct  1022  WHGLACKQFLAGYRAEVDGCPSVPADDAAFEELLNLFLLEKALYEVCYEAANRPDWLAIP  1081
```

```
Query  476   LAGVHRLLE  484
             + GV  +LE
Sbjct  1082  VGGVLGVLE  1090


> MKNNJHOE_00428
Length=1101

 Score = 399 bits (1026),  Expect = 2e-131, Method: Compositional matrix adjust.
 Identities = 222/462 (48%), Positives = 291/462 (63%), Gaps = 12/462 (3%)

Query  32    QRYALPLALAWESASDDPLARLQAQVVARVRRGSHLGVLYDATASDELPRLLIDAIRGGA  91
             QRY LPL++AWE+ASDDPL+RL    +AR R G H+G ++D  A+  L R +I A+
Sbjct  636   QRYLLPLSIAWETASDDPLSRLLPFTLARARTGGHIGAIHDGMATPALSRAVIRAVAQER  695

Query  92    ELPTRGGARLVCRSTGALTA-YSDVNTSN---CRRLGVEQSNTSMLIDDQMIIKLYRRLQ  147
             E+PTR G RL  R T A +   +D   +       RLG EQSNTS+LI + +I K  RR++
Sbjct  696   EVPTRSGGRLEFRKTKAFSQELADAAIAGELPVERLGREQSNTSVLIGEDVIFKALRRVE  755

Query  148   SGIHPEVEIGHFLTSVAGYTSAPALLGSVELLDDDGSVTAIAVLQEFVRNQGDGWEMTLD  207
             SG+HPE+EIG FLT VA + +AP LLGSVE+ D  G  TAI VLQ FVRNQGDGW  TLD
Sbjct  756   SGVHPELEIGRFLTDVASFNNAPPLLGSVEMRDAXGRPTAIGVLQGFVRNQGDGWGFTLD  815

Query  208   HLERVLNQLDLTLLSAEDYDD-EAAHESYWASVATLARRVGELHLAFALTVDDPAFAPEP  266
             +L+RVL QL+   L A+ + + E  H  Y A       R+G+LH AFA+ V+DPAF PEP
Sbjct  816   YLDRVLGQLE--FLPADVHPEREDLHVMYLALAGNAGERIGQLHQAFAMPVEDPAFRPEP  873

Query  267   TSEDDIATWNRQIAELATKAHRILESAVHADALGEEARSLGTQLLASWPAIEALATIPAT  326
                 D  +W   + E A  A   L  A  A  +  E R    QLL +WP I A+ +
Sbjct  874   ADPADAESWTASVLEQAEGARTALNRAP-AGEMTSETRLDIEQLLNAWPQITAVTSEMRA  932

Query  327   TLTGIMKTRIHGDLHLGQVVVAGTNFFILDFEGEPLRTLEARRQXSFPLRDVAGLLRSFD  386
              + G +KTR+HGDLHLGQVV+   +F+ILDFEGEP+R +  RR    PL+DVAG+LRSFD
Sbjct  933   HVHGALKTRVHGDLHLGQVVIVREDFYILDFEGEPMRPMAERRIKQSPLKDVAGMLRSFD  992

Query  387   YAGSAALIRRGSI-ANAEKGVASERDAITKWRRETSARFMSTYRETVADCAALPQAEEAF  445
             YA  AAL+    + A   + +  E +A   WR+ T  RF++ YR+    CA++P  +  F
```

```
Sbjct  993   YAAHAALLSHPEVRAPVRERLQQEMEA---WRKLTIERFVAGYRQATEGCASVPTDDVTF  1049

Query  446   VAALDAFVIEKALYEICYEAANRPDWLSIPLAGVHRLLETVR  487
                LD F++EK LYEI YEAANRPDWL+IP+ G  RLL ++R
Sbjct  1050  EQLLDLFLLEKVLYEIRYEAANRPDWLAIPVTGAIRLLASLR  1091


 Score = 22.3 bits (46),  Expect = 0.76, Method: Compositional matrix adjust.
 Identities = 9/18 (50%), Positives = 13/18 (72%), Gaps = 0/18 (0%)

Query  181  DDGSVTAIAVLQEFVRNQ  198
            DDG+V   A+L +F+ NQ
Sbjct  576  DDGAVLWHAILPDFLPNQ  593


> AMCANFMH_01079
Length=1090

 Score = 328 bits (841),  Expect = 8e-105, Method: Compositional matrix adjust.
 Identities = 195/461 (42%), Positives = 259/461 (56%), Gaps = 17/461 (4%)

Query  32    QRYALPLALAWESASDDPLARLQAQVVARVRRGSHLGVLYDATASDELPRLLIDAIRGGA  91
               Y LPLA+AWE+ +D+P  R     ++++R G H G++YD  A     R ++ A+R
Sbjct  641   HHYLLPLAIAWETPTDEP-TRFLPFALSKIRSGPHSGLIYDGYADAGYMRAVLAALRDAR  699

Query  92    ELPTRGGARLVCRSTGALTAYSDVNTSNCRRLGVEQSNTSMLIDDQMIIKLYRRLQSGIH  151
              +P   G  +  + TG L           R LGVEQSNTS++I +Q++ K YRRL  G+H
Sbjct  700   VIPASSG-EVHFKPTGKLQDVVFDGEPEVRALGVEQSNTSIIIGEQIVAKGYRRLHRGVH  758

Query  152   PEVEIGHFLTSVAGYTSAPALLGSVELLDDDGSVTAIAVLQEFVRNQGDGWEMTLDHLER  211
              EVE+  +LT VAGY + P LLGSVE  D DG+ TA+ +LQ FVRNQGDGW+ TL HL+
Sbjct  759   LEVEVARYLTDVAGYANTPPLLGSVEHRDVDGTPTALCILQGFVRNQGDGWKYTLHHLKG  818

Query  212   VLNQLDLTLLSAEDYDDEAA-----HESYWASVATLARRVGELHLAFALTVDDPAFAPEP  266
             +L +      SA D  D           Y   + TL  R GELH AF L   DPAF PEP
Sbjct  819   LLER------SATDEPDPEGGLPNVDRIYDVLIETLGVRTGELHQAFGLQTGDPAFEPEP  872
```

```
Query  267   TSEDDIATWNRQIAELATKAHRILESAVHADALGEEARSLGTQLLASWPAIEALATIPAT  326
               +  D+  W  Q+  LA +A   LE A   D L  E   +   +L+   A+  L +
Sbjct  873   ITTQDLQGWREQVLGLAQRALGELEQA--RDGLPPETHDMVDAVLSEAGALGPLISGLVP  930

Query  327   TLTGIMKTRIHGDLHLGQVVVAGTNFFILDFEGEPLRTLEARRQXSFPLRDVAGLLRSFD  386
               T   KTR HGD HLGQV++   ++ I+DFEGEPLR LE RR    PL+DVAG+LRSFD
Sbjct  931   ESTSATKTRYHGDYHLGQVLLGMDDWMIIDFEGEPLRPLEERRSKHCPLKDVAGMLRSFD  990

Query  387   YAGSAALIRRGSIANAEKGVASERDAITKWRRETSARFMSTYRETVADCAALPQAEEAFV  446
             YA  AAL    +  +  +     R  +  W+++ S  F+S YR  VA C++ P
Sbjct  991   YAAWAALFA--ATEDRPEDFEPLRPVVEAWKQQASETFLSGYRRAVAGCSSYPTDATEAD  1048

Query  447   AALDAFVIEKALYEICYEAANRPDWLSIPLAGVHRLLETVR  487
             A LD F++EKA YEI YE  NRP W+ IPL G+HR+L   R
Sbjct  1049  ALLDLFLVEKAAYEIRYELGNRPSWVRIPLLGLHRILTARR  1089



Lambda      K        H        a         alpha
   0.319    0.133    0.385    0.792     4.96

Gapped
Lambda      K        H        a         alpha    sigma
   0.267   0.0410    0.140     1.90     42.6     43.6

Effective search space used: 4129965


  Database: treS_defluviivoccus.faa
    Posted date:  Nov 16, 2021  8:06 AM
  Number of letters in database: 9,915
  Number of sequences in database:  10



Matrix: BLOSUM62
Gap Penalties: Existence: 11, Extension: 1
```

Neighboring words threshold: 11
Window for multiple hits: 40
